# Validation of a prognostic model for chronic obstructive pulmonary disease (COPD) and evaluation of methods to include related data

**DOI:** 10.1186/s41512-026-00245-8

**Published:** 2026-11-06

**Authors:** Haya Elayan, Jonathan Broomfield, Mark J. Rutherford, Matthew Sperrin, Glen P. Martin, David A. Jenkins, Sarah Booth

**Affiliations:** 1 Division of Informatics, Imaging and Data Science, Faculty of Biology, Medicine and Health, University of Manchester, Manchester, UK; 2 Division of Public Health and Epidemiology, School of Medical Sciences, University of Leicester, Leicester, UK; 3 Department of Respiratory Sciences, University of Leicester, Leicester, UK

**Keywords:** COPD, Prognostic models, Temporal and regional validation, Related data

## Abstract

**Background and objectives:**

Prognostic models estimate a patient’s individual risk of future health outcomes using clinical and demographic data, supporting treatment planning. Such models are particularly useful for chronic diseases, where predicting disease progression can help guide interventions and improve patient outcomes. In chronic obstructive pulmonary disease (COPD), several models have been developed to predict long-term mortality risk. However, these models are often trained on patients diagnosed 10 to 20 years ago, and outcomes may have improved since then. As a result, such models may be miscalibrated by the time they are published, potentially limiting their clinical utility.

**Methods:**

We aim to validate an existing COPD prognostic model for five-year mortality from the date of diagnosis to explore potential miscalibration over time and across regions. Moreover, we evaluated modelling methods to improve predictive performance in target datasets by incorporating related (ancillary) data while accounting for heterogeneity, including: (i) developing a logistic regression model in the target data, (ii) Intercept Recalibration to update the intercept using the target data, (iii) Logistic Recalibration to update both intercept and calibration slope using the target data, and (iv) Membership-based recalibration that reweights ancillary data using propensity scores. These approaches were assessed using newly collected target datasets, or by leveraging the existing development dataset only.

**Results and conclusion:**

The existing model demonstrated similar discrimination across the external validation settings, with mean AUC values ranging from 0.79 (95% confidence interval [CI] 0.72 to 0.85) to 0.80 (95% CI 0.78 to 0.83). However, mean calibration-in-the-large (CITL) values varied from 0.001 (95% CI −0.25 to 0.34) to 0.46 (95% CI 0.29 to 0.61) in the newly available target data, likely reflecting shifts in baseline risk. Among the methods evaluated for improving prediction by incorporating ancillary data while accounting for heterogeneity, Intercept Recalibration was a simple method that generally reduced CITL to near-zero levels. Importance weighting using propensity scores also provided a potential alternative, although its performance varied across settings. These methods can be applied during development using existing data or after development with newly collected data, and may improve model calibration for the target population.

**Supplementary Information:**

The online version contains supplementary material available at https://doi.org/10.1186/s41512-026-00245-8.

## Background

Chronic Obstructive Pulmonary Disease (COPD) is a progressive lung disease that primarily affects the ability to breathe. It is a long-term condition characterized by persistent airflow limitation and inflammation of the airways [1]. The prevalence of COPD in the UK is estimated to be around 1–2% [2]. The symptoms of COPD often develop gradually and worsen over time. They include shortness of breath, chronic cough, wheezing, chest tightness, frequent respiratory infections, fatigue, and unintended weight loss. As the condition progresses, everyday activities become increasingly difficult, impacting a patient’s quality of life. Diagnosis of COPD involves medical history evaluation, physical examination, lung function tests like spirometry, and imaging studies. Treatment focuses on managing symptoms, preventing exacerbations, and slowing down the progression of the disease. It typically involves a combination of medications (bronchodilators, steroids), pulmonary rehabilitation, oxygen therapy, and lifestyle changes such as smoking cessation and avoiding lung irritants [3].

Prognostic models aim to predict the risk of an event occurring in a particular population (target population) with a disease, based on relevant clinical information [4–6]. Prognostic models for COPD therefore often include usual predictors of health differences, such as age, gender and body mass index (BMI), alongside disease-specific predictors like forced expiratory volume in 1 second (FEV1% predicted). Models such as these can be used by health practitioners to administer care, assist with disease planning and management and (in some instances) prescribe treatments to patients at an elevated risk [7, 8].

Previous COPD prognostic models have used mortality at either 1, 5, or 10 years from the date of COPD diagnosis as an outcome [9–11]. However, the models with longer-term outcomes, such as 5-year or 10-year mortality, require an extended follow-up such that patients diagnosed a number of years ago are included in model development. If survival has changed over time—due to improvements in treatment, healthcare delivery, or other factors—this can cause the model to give miscalibrated predictions that overestimate or underestimate the risk of dying in a more recent target population (or a geographically different target population) [12, 13]. For example, Tan et al. [14] attributed such variations in COPD mortality rates across different countries or regions, as well as over time, to differences in healthcare systems, database infrastructures, and coding practices—factors that are likely to influence observed changes.

One solution to this problem is to develop the model using data from the particular population, period, or setting for which prediction is of primary interest, such as a recent cohort or a specific region or healthcare setting. We refer to these data as the **target data**. However, this approach has limitations when the target dataset has a small sample size, which may increase the risk of overfitting during model development [15–18]. Related data from another population, period, or healthcare setting may also be available to support model development, which we refer to as the **ancillary data**. Together, these terms form the target–ancillary framework used in this study and describe the relationship of the available data to the population, period, or setting for which prediction is of primary interest. Both target and ancillary data may contribute to model development. We use development data as the conventional overarching term for all observations used to derive a model or estimate its parameters. Depending on the modelling approach, the development data may comprise target data, ancillary data, or both. For example, when developing a COPD risk model for application in Greater Manchester, data from Greater Manchester would constitute the target data, while related data from other regions of the UK could constitute the ancillary data. However, there can be a degree of heterogeneity between the target and the ancillary datasets, which reflects differences in their underlying distributions. These differences may occur due to data distribution shift which arise as a result of case-mix shift (changes in the distribution of predictors), event rate shift (changes in outcome prevalence), or predictor-outcome association shift (changes in relationships between predictors and outcome prevalence). This heterogeneity can negatively impact model performance, leading to inaccurate predictions [19–22]. Therefore, when incorporating an ancillary dataset, it is crucial to account for the heterogeneity between the ancillary and target datasets to improve the predictive performance of the prognostic model.

Therefore, this study aims to validate an existing COPD prognostic model, originally developed using data from Clinical Practice Research Datalink (CPRD), which predicts a binary five-year mortality outcome from the date of COPD diagnosis. The validation will be performed across multiple target datasets to assess potential miscalibration due to temporal or regional variations. In addition, various modelling methods will be assessed for their potential to improve predictive performance by incorporating ancillary datasets, while accounting for heterogeneity between the target and ancillary datasets. Methods to be used are logistic regression (developed on data from the target only), logistic and intercept regression updating methods (developed on the ancillary data only or ancillary and target data, then updated to the target) [6, 23, 24], and importance weighting using propensity scores (developed on all available data from the ancillary and target, while weighting the ancillary data samples based on their similarity to the target) [25].

## Methods

In this section, we describe the replication of an existing COPD prognostic model and its external validation across various target datasets. We then evaluate different modelling methods for their potential to enhance predictive performance by incorporating ancillary data while accounting for differences between the target and ancillary datasets. Finally, we evaluate whether applying these methods to a temporal split of the original development data during model development improves predictive performance in a later sample from the same target population.

### Dataset, cohort construction, and outcome

Data from CPRD GOLD were utilised, including patients diagnosed with COPD. We aimed to replicate the prognostic model previously developed by Kiddle et al. [10]. Diagnosis was determined using previously validated COPD codes [12], which are provided in the Appendix and were also used by Kiddle et al. This model was selected because (almost) the same data were available, it predicts a binary mortality outcome at five years from diagnosis, and there is an opportunity to assess potential miscalibration given the timing of the study.

Although we applied the same inclusion criteria, exact replication of the original cohort was not feasible due to differences in the structure and availability of the data at the time of extraction. In particular, variation in the availability and coding of percent predicted forced expiratory volume in one second (FEV1% predicted) and BMI resulted in higher missingness in our dataset than reported by Kiddle et al. (see Additional file, section S1 for details). We therefore constructed the closest comparable cohort available within CPRD, defined as one matching the original study’s eligibility criteria, time period, and demographic profile as closely as possible given these constraints. We also used more recent and setting-specific datasets to assess potential miscalibration; details of these study settings and dataset definitions are provided in Section “Study settings and the roles of the ancillary and target datasets”. A total of 55,593 patients diagnosed between 1 January 2004 and 19 September 2012 were included in the reproduced cohort. The cohort covers data from 9 practice regions across England and 3 other regions outside of England (Wales, Scotland, and Northern Ireland). These 3 regions were not included because we restricted to the CPRD ‘linked group’ (English practices consenting to linkage with ONS mortality and IMD data), in line with the linkage approach used by Kiddle et al.

Full details of cohort construction, variable coding, and differences from the original study are provided in the Additional file S1. Also, details on the performed Patient and Public Involvement (PPI) are provided in the Additional file S2.

### Reproducing an existing COPD model: The candidate model

Consequently, we reproduced the original prognostic model developed by Kiddle et al. using logistic regression, applying the same covariates and functional form in the full reproduced cohort (all regions; 1/1/2004 to 19/9/2012). We refer to this reproduced model as the **“candidate model”**. The model structure and analytical approach were replicated within the constraints of the available data. The CPRD was linked to the Office for National Statistics (ONS) data to obtain accurate dates of death, enabling alignment with the original study. Additional linkage to Hospital Episode Statistics (HES) allowed us to identify certain covariates used in the Kiddle et al. The outcome of interest was defined as binary five-year mortality from the date of COPD diagnosis, consistent with Kiddle et al.’s model. Because 5-year vital status (death within five years of COPD diagnosis) was ascertained for all included patients using ONS-linked mortality in the CPRD linked group, the outcome could be defined as a complete binary variable, making logistic regression an appropriate modelling choice. Although the outcome is described as 5-year mortality, the reproduced modelling framework follows the original Kiddle et al. specification and is most naturally interpreted in terms of 5-year survival.

A large proportion of missing data were observed for FEV1% predicted and BMI. To handle this, we followed Kiddle et al.’s approach by both imputing missing values and including missingness indicators. Specifically, missing values for continuous covariates such as BMI and FEV1% predicted were imputed using the median observed value within the dataset. In addition, dichotomous variables were created and included in the model for BMI and FEV1% predicted to indicate whether the original measurement was missing for a patient, capturing any potential informative missingness. For the categorical variable smoking status, missing values were imputed by random sampling from the observed non-missing values, preserving the variable’s marginal distribution. Also following the Kiddle et al.’s approach, age, BMI, and FEV1% predicted were mean-centred, and their squared terms were included as covariates in the model.

This reproduced modelling framework was then used to derive the candidate model within each study setting, as defined in the next section. The protocol for this study, which is covered by the CPRD Independent Scientific Advisory Committee ethics approval, is provided here and in Additional File-CPRD_Protocol.

### Study settings and the roles of the ancillary and target datasets

The study considers four settings defined by temporal and/or regional comparisons. In each setting, the target and ancillary data were specified according to the comparison of interest. These terms describe the relationship of the available data to the population, period, or setting for which prediction is of primary interest (Table 1). Depending on the modelling approach, the development data may comprise target data, ancillary data, or both. The setting-specific datasets were used to externally validate the candidate model or, where appropriate, to fit and evaluate setting-specific variants of the candidate-model specification and the alternative modelling methods described later. In this section, we outline the four settings and the corresponding roles of the ancillary and target data.

1. **Temporal–New (Temporal setting - New data; target data for patients diagnosed 20/9/2012 to 30/12/2014):** The candidate model was fitted on the full reproduced cohort of patients diagnosed between 1/1/2004 and 19/9/2012. Within this temporal comparison, this development dataset constitutes the ancillary data (55,593 samples). The candidate model was externally validated on a more recent dataset of patients diagnosed between 20/9/2012 and 30/12/2014, which constitutes the target data (10,907 samples) and was extracted using the same inclusion criteria as the reproduced cohort.
2. **NorthEast–New (Temporal and Regional setting - North East; target data for patients diagnosed 20/9/2012 to 30/12/2014 from North East Region):** A regional variant of the candidate-model specification was fitted on patients diagnosed between 1/1/2004 and 19/9/2012 from all practice regions except the North East. Within this comparison, these development data constitute the ancillary data (53,728 samples). The model was externally validated on a more recent dataset of patients diagnosed between 20/9/2012 and 30/12/2014 from the North East region, which constitutes the target data (235 samples) and was extracted using the same inclusion criteria as the reproduced cohort. This region was selected to assess potential heterogeneity in a target dataset of limited sample size.
3. **London–New (Temporal and Regional setting - London; target data for patients diagnosed 20/9/2012 to 30/12/2014 from London Region)**:
  A regional variant of the candidate-model specification was fitted on patients diagnosed between 1/1/2004 and 19/9/2012 from all practice regions except London. These development data constitute the ancillary data (50,985 samples). The model was externally validated on a more recent dataset of patients diagnosed between 20/9/2012 and 30/12/2014 from the London region, which constitutes the target data (1,492 samples) and was extracted using the same inclusion criteria as the reproduced cohort. This setting used a different region as the target to broaden the assessment across geographical settings and target dataset sample sizes.
4. **Temporal–Existing (Temporal setting - Existing data; target data for patients diagnosed 1/1/2010 to 19/9/2012):**
  This setting assessed whether the temporal structure of the original development data could be used to develop models that were better aligned with the target population at deployment. The candidate model was fitted on the full reproduced cohort of patients diagnosed between 1/1/2004 and 19/9/2012, consistent with the Temporal–New setting, and was externally validated on the newly available cohort of patients diagnosed between 20/9/2012 and 30/12/2014 (10,907 samples). For the methods described in Section “Methods to improve predictive performance in target datasets”, the same development data were divided using a temporal cut-off. The ancillary data consisted of patients diagnosed between 1/1/2004 and 31/12/2009 (39,068 samples), while the target data consisted of patients diagnosed between 1/1/2010 and 19/9/2012 (16,525 samples). The target data were drawn from the most recent period of the available development data, under the assumption that they were most relevant to the target population at model deployment. The resulting models were also externally validated on the newly available cohort of patients diagnosed between 20/9/2012 and 30/12/2014. These data came from a later sample of the same target population and did not contribute to model development. This setting therefore allowed us to examine whether applying the modelling methods to a temporal split of the original development data could begin to address potential calibration drift during model development. More specifically, it provided insight into whether a more up-to-date model could be developed from the outset, without initially relying on entirely new target data. Further details on the application and evaluation of the methods are provided in Section “Analysis plan”.

**Table 1:** Definitions of data-related terms used in this study

Term	Definition in this study
Target data	Data from the population, period, or setting for which prediction is of primary interest. Depending on the analysis, these data may be used for model fitting, recalibration, and/or evaluation.
Ancillary data	Related data from another population or setting that are available to support model development. These data may contribute directly to model fitting.
Development data	The overarching term for all data used to derive a model or estimate its parameters. Depending on the modelling approach, the development data may comprise target data, ancillary data, or both.
External validation data	Target data used only to assess model predictive performance and not used for model development or recalibration.

### Methods to improve predictive performance in target datasets

The following modelling methods were used to develop models for the different target datasets by incorporating ancillary data while accounting for differences between the target and ancillary datasets.

#### Target-only logistic regression

This method uses the standard unweighted logistic regression to develop a new model on the target data only.

#### Intercept recalibration

This Re-calibration updating method uses the new available data to update an existing model by updating the intercept only, which intends to correct “calibration-in-the-large” [6, 24, 26]. We implement this method in two variations as follows:

1. **Intercept Recalibration-all data**: We first develop a logistic regression model using all development data (both ancillary and target), then we apply the model to the target data to obtain the linear predictor ($LP_{target}$) for all individuals. We then update the model by fitting a logistic regression on the target data with $LP_{target}$ as an offset variable (the regression coefficient is constrained to be 1). This variation is conceptually similar to the temporal recalibration approach proposed by Booth et al. [27, 28] for survival models, in that it updates the baseline to reflect a more recent time period. In our logistic regression setting, this corresponds to recalibrating the baseline risk (outcome prevalence), rather than a baseline (cumulative) hazard.
2. **Intercept Recalibration-ancillary only**: We first develop a logistic regression model on ancillary data only, then we apply the model to the target data to obtain the linear predictor ($LP_{target}$) for all individuals. Next, we update the model by fitting a logistic regression on the target data with $LP_{target}$ as an offset variable (the regression coefficient is constrained to be 1).

#### Logistic recalibration

This Re-calibration updating method uses the new available data to update an existing model by updating both the intercept and the overall calibration slope [6, 24, 26]. We implement this method in two variations as follows:

1. **Logistic Recalibration-all data**: We first develop a logistic regression model using all development data (both ancillary and target), then we apply the model to the target data to obtain the linear predictor ($LP_{target}$) for all individuals. Next, we fit a logistic regression on the target with setting $LP_{target}$ as the only variable. Similar to method 2 in Vergouwe et al. [24].
2. **Logistic Recalibration-ancillary only**: We first develop a logistic regression model on the ancillary data only, then we apply the model to the target data to obtain the linear predictor ($LP_{target}$) for all individuals. Next, we fit a logistic regression on the target with setting $LP_{target}$ as the only variable.

#### Membership-based recalibration

This method develops a weighted model by giving individuals from the ancillary data higher weights when they are ‘relevant’ to target and lower weights when they are ‘less relevant’ to target [25]. The weights are defined based on the relatedness of the individual samples from the ancillary data compared with the target data using the **Membership model**- a probabilistic similarity metric- that is based on the propensity score, thus adjusting the distribution of the ancillary data to correct for distributional changes [19]. The membership propensity score is defined as the conditional probability of an individual being a member of the ancillary dataset, given its observed values from a set of predictor variables [29–32].

We first calculate the weights of the ancillary dataset using the membership based weighting scheme using the same set of predictors used in the prediction model, while weighting the target dataset samples as 1. Following that, we adjust the weights using an overall forgetting factor by multiplying them by the ratio of sample sizes (i.e., the sample size of the ancillary dataset divided by the sample size of the target dataset) to account for differences in sample sizes between the target and the ancillary datasets. Finally after developing the weighted model, we adjusted the calibration in the large (CITL) of the weighted model through adding a dummy variable for the target membership.

### Analysis plan

The settings and corresponding target/ancillary datasets are defined in Section “Study settings and the roles of the ancillary and target datasets”, and the modelling methods are defined in Section “Methods to improve predictive performance in target datasets”. This section clarifies how each analysis is applied and evaluated within the four settings.

- **Candidate model:** The candidate model, defined in Section “Methods to improve predictive performance in target datasets” as the reproduced Kiddle model fitted on the full reproduced cohort, was externally validated in the Temporal–New and Temporal–Existing settings.
- **Regional candidate-model variants:** In the NorthEast–New and London–New settings, regional variants of the candidate-model specification were fitted after excluding the target region from the ancillary dataset, and were then externally validated on the corresponding target dataset.
- **Alternative modelling methods in new available target-data settings (Temporal–New, NorthEast–New, London–New):** For the alternative modelling methods, the target dataset contributes to model fitting and/or recalibration. Therefore, performance is evaluated within the same target dataset using internal validation with optimism adjustment (Section “Performance metrics”).
- **Alternative modelling methods in the existing development-data setting (Temporal–Existing):**
  The alternative modelling methods were applied during model development using the ancillary data (1/1/2004–31/12/2009) and/or target data (1/1/2010–19/9/2012) defined in Section “Study settings and the roles of the ancillary and target datasets”, depending on the method. The resulting models were evaluated within the 1/1/2010–19/9/2012 target data using optimism-adjusted internal validation and were also externally validated using data from the subsequent period. Their performance was compared with that of the candidate model, particularly using data from the subsequent period, to assess whether applying the alternative methods during model development may have reduced the potential effect of temporal heterogeneity within the original development data.

**Fig. 1:**
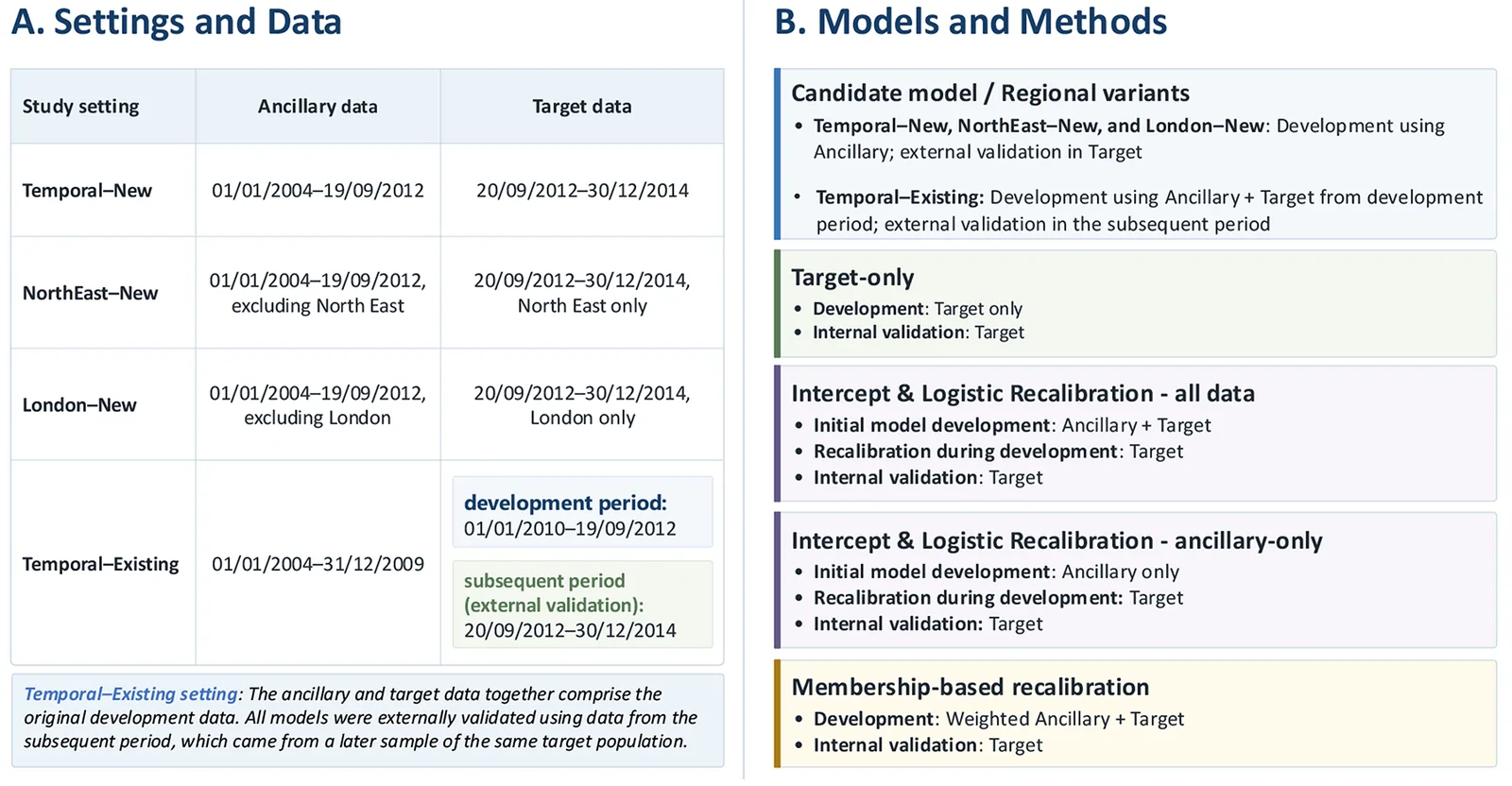
Overview of analysis plan across settings

Figure 1 provides a schematic overview of the analysis plan, including the datasets and modelling methods applied within each setting

### Performance metrics

For the candidate model, external validation performance was summarised in the setting-specific target datasets. For the methods used to improve model performance, both apparent and bootstrap internal validation results of predictive performance (AUC, calibration slope (CSLOPE) and calibration-in-the-large (CITL), and Brier Score) were reported from the target dataset. The adjusted optimism for each performance metric was estimated to provide an estimate of predictive performance after removing in-sample optimism as developing a model in a dataset and then testing the model in that same data (without adjustment) will lead to overly optimistic performance estimates [6, 26].

The adjusted optimism for each performance metric was estimated by calculating the difference between the bootstrap apparent metric and bootstrap test metric of the 200 bootstrap samples, then subtracting the mean optimism over 200 bootstrap samples from the development apparent metric. Also, the 95% confidence interval (CI) was calculated for the bootstrap apparent and bootstrap test for each performance metric across the 200 bootstrap iterations. For external validation, performance metrics were similarly estimated by bootstrapping the external validation dataset 200 times, and are reported as the mean and 95% confidence interval across the bootstrap samples.

### Software

Data analysis was performed in R version 4.3.1, while data preparation was performed in both R version 4.3.1 and Stata version 18. Code to replicate the analysis can be found in the following GitHub repository: (https://github.com/HayaElayan/Validation-of-a-Prognostic-Model-for-COPD-and-Evaluation-of-Methods-to-Include-Related-Data)

## Results

We first describe characteristics of the reproduced cohort and compare predictor effects between Kiddle et al.’s model and reproduced model. We then report external validation results for the candidate model and its regional variants across the study settings. Next, we present results for the alternative modelling methods in the settings with newly collected target data. Finally, we report results for the Temporal–Existing setting, including internal validation within the development-period split and the additional external validation using data from the subsequent period.

### Reproduced cohort characteristics

The reproduced cohort’s demographics and clinical characteristics are shown in Table 2 in comparison with the original Kiddle’s group reported cohort. The main difference was the missingness of BMI and FEV1% predicted as explained in the dataset Section “Reproducing an existing COPD model: the candidate model” and in the Additional File, section S1.

**Table 2:** Demographics and clinical characteristics of reported vs reproduced cohorts

Variables	Reported (*N* = 60,060)	Reproduced (*N* = 55,593)
Sex		
Male	31,582 (53%)	29,277 (53%)
Female	28,478 (47%)	26,316 (47%)
Age, years	68 (59 - 76)	68 (60 - 76)
BMI, kg/m2	26 (23 - 30)	26 (23 - 30)
Not recorded	5,824 (10%)	18,922 (34%)
Smoking		
Never smoker	8,853 (15%)	7409 (13%)
Ex smoker	24,488 (41%)	23,406 (42%)
Current smoker	25,632 (43%)	24,161 (43%)
Not recorded	1,087 (2%)	617 (1%)
FEV1% predicted	65 (50 - 79)	63 (49 - 76)
FEV1% predicted not recorded	24,691 (41%)	38,219 (69%)
Deaths within 5 years of COPD diagnosis	14,139 (24%)	13,034 (23%)

**Fig. 2:**
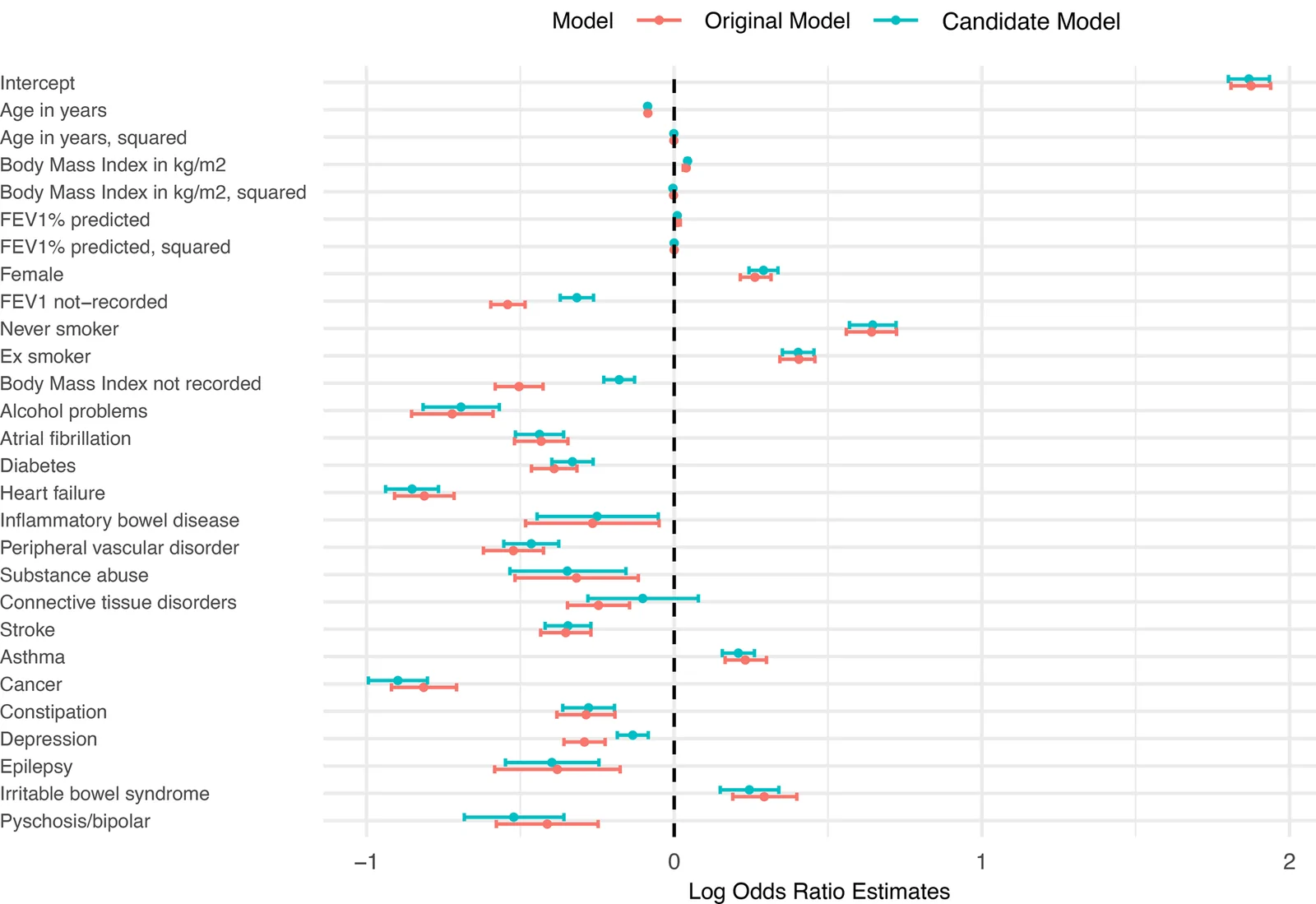
OR estimates for the original Kiddle et al.’s model vs candidate model. The candidate model fitted on the full reproduced cohort

Figure 2 shows the odds ratio estimates for the original Kiddle et al.’s model and the candidate model fitted on the full reproduced cohort, these are expressed with respect to 5-year survival. Variations in the availability and coding of BMI and FEV1% predicted, which resulted in higher missingness in our dataset, are likely to explain much of the variation observed between the odds ratios from our candidate model and those from Kiddle et al.’s model.

### External validation of the candidate model and regional variants

The candidate model and its regional variants demonstrated similar discrimination across the validation settings, as shown in Table 3. However, CITL values greater than zero highlight challenges in maintaining calibration when validating the model on more recent data, particularly in the temporal setting, likely reflecting changes in outcome prevalence over time.

**Table 3:** External validation of the candidate model and regional candidate-model variants across target datasets

Setting	AUC	Calibration-in-the-large	Calibration slope	Brier Score
Temporal–New & Temporal–Existing	0.793(0.782, 0.802)	0.283(0.228, 0.343)	0.994(0.940, 1.043)	0.134(0.130, 0.138)
NorthEast–New	0.786(0.719, 0.849)	0.001(−0.254, 0.341)	1.010(0.696, 1.350)	0.147(0.120, 0.172)
London–New	0.803(0.778, 0.828)	0.455(0.288, 0.613)	0.934(0.815, 1.044)	0.128(0.117, 0.138)

For Temporal–New and Temporal–Existing, results are from the candidate model fitted on the full reproduced cohort, and therefore share the same external-validation results. *∘* For NorthEast–New and London–New, results are from regional variants of the candidate-model specification fitted after excluding the target region from the ancillary dataset

### Performance of the modelling methods using new available target datasets

#### Temporal–New

**Fig. 3:**
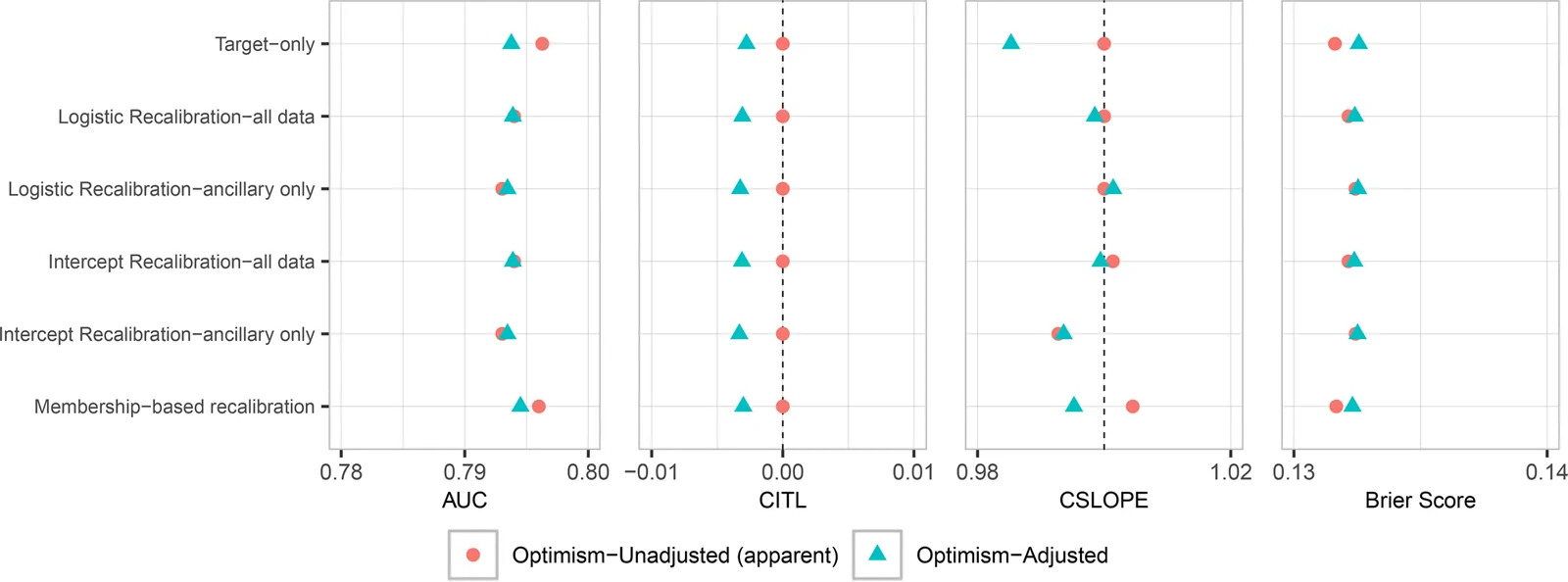
Internal validation-adjusted and unadjusted performance metrics: temporal–New *∙* candidate model external validation results on same target dataset are AUC: 0.793(0.782, 0.802), CITL: 0.283(0.228, 0.343), CSLOPE: 0.994(0.940, 1.043) , Brier score: 0.134(0.130, 0.138)

As shown in Fig. 3, all models achieved an optimism-adjusted AUC similar to the candidate model, around 0.79. In terms of calibration, all models achieved optimism-adjusted calibration-in-the-large values of −0.003, compared with an external-validation mean CITL value of 0.283 for the candidate model, and had calibration slopes closer to 1. When expressed as the difference between expected and observed survival (Table 4), the candidate model showed a 3.0 percentage point miscalibration, indicating overestimation of risk (or underestimation of survival). Additionally, all models achieved slightly lower Brier scores than the candidate model, indicating better overall predictive performance. Moreover, as illustrated in Figure S1 in the Additional File, the Target-only and Membership-based models showed higher bootstrap apparent and test AUC performance than the other models. In the bootstrap test calibration slope, the Target-only model and both Logistic Recalibration models had wider confidence intervals (CIs) (across 200 iterations) compared to the other models. In contrast, both Intercept Recalibration models had wider CIs for the bootstrap apparent calibration slope.

**Table 4:** Expected and observed survival across external validation datasets for the candidate model and regional candidate-model variants

Setting	Observed survival (%)	Expected survival (%)	Difference between Expected and Observed survival (% point)
Temporal–New & Temporal–Existing	79%	76%	−3.0% points
NorthEast–New	75%	75%	0.04% points
London–New	81%	75%	−5.8% points

For Temporal–New and Temporal–Existing, results are from the candidate model fitted on the full reproduced cohort, and therefore share the same external-validation results. *∘* For NorthEast–New and London–New, results are from regional variants of the candidate-model specification fitted after excluding the target region from the ancillary dataset

#### NorthEast–New

**Fig. 4:**
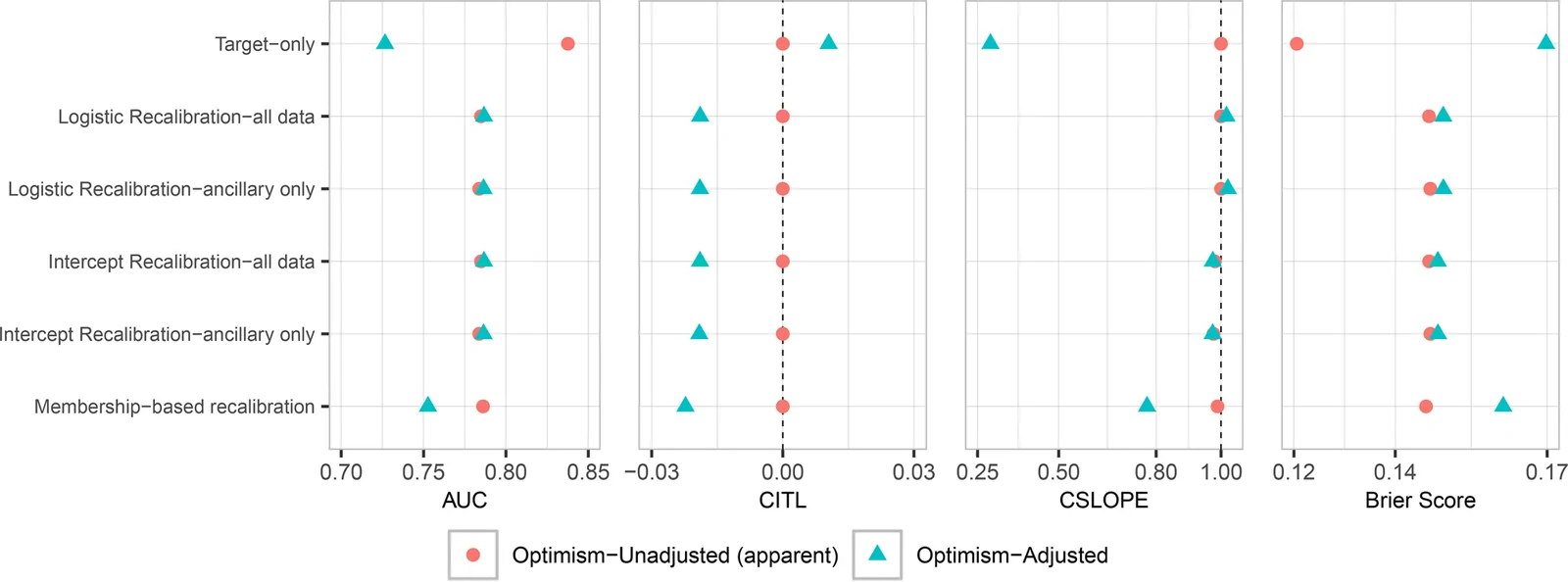
Internal validation-adjusted and unadjusted performance metrics: NorthEast–New *∙* candidate model external validation results on same target dataset are AUC: 0.786(0.719, 0.849) , CITL: 0.001(−0.254, 0.341), CSLOPE: 1.010(0.696, 1.350), Brier score: 0.147(0.120, 0.172)

As illustrated in Fig. 4, all alternative models had optimism-adjusted calibration-in-the-large estimates close to zero, at approximately −0.02. When expressed as the difference between observed and expected survival as shown in Table 4, the candidate model showed only a 0.04 percentage point difference, supporting accurate estimation of risk. Furthermore, all Intercept and Logistic Recalibration models demonstrated slightly higher optimism-adjusted AUCs than the candidate model. In contrast, the Membership-based recalibration model had a slightly lower AUC of 0.753, while the Target-only model showed a notably lower AUC of 0.727 compared to both Recalibration models and the candidate model. The Membership-based recalibration and Target-only models had optimism-adjusted calibration slope estimates of 0.772 and 0.29, respectively. These lower estimates may reflect instability associated with the effective sample size and, for the Membership-based recalibration model, the additional down-weighting of the ancillary data. The Target-only and Membership-based models demonstrated better bootstrap apparent AUC performance compared to the other models. However, both models had wider bootstrap test AUC and calibration slope CIs, as shown in Figure S2 in Additional File. Additionally, both Intercept Recalibration models had smaller bootstrap test calibration slope CIs compared to the other methods. However, both models showed wider CIs for the bootstrap apparent calibration slope.

#### London–New

**Fig. 5:**
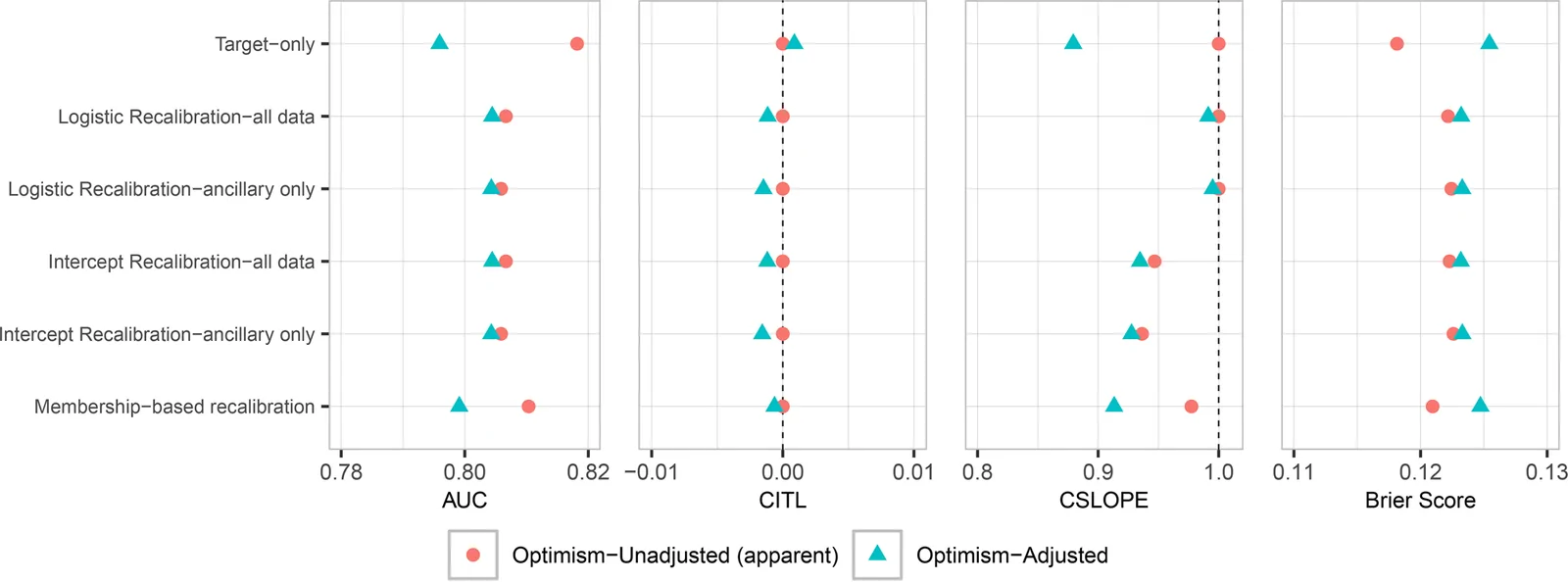
Internal validation-adjusted and unadjusted performance metrics: London–New *∙* candidate model external validation results on same target dataset are AUC: 0.803(0.778, 0.828), CITL: 0.455(0.288, 0.613), CSLOPE: 0.934(0.815, 1.044), Brier score: 0.128(0.117, 0.138)

Consistent with the Temporal–New setting, all models improved calibration, achieving optimism-adjusted calibration-in-the-large values closer to zero compared with the candidate model, which had an external-validation mean CITL value of 0.455, as shown in Fig. 5. When expressed as the difference between expected and observed survival (Table 4), the candidate model showed a 5.8 percentage point miscalibration, indicating overestimation of risk (or underestimation of survival). Both Logistic Recalibration models had optimism-adjusted calibration slope estimates closest to 1 among the models evaluated, while the remaining models—including the candidate model—showed comparable but slightly lower slopes. Additionally, all models achieved slightly better Brier scores than the candidate model, which had a mean value of 0.128, suggesting improved overall predictive performance. In this setting also, the Target-only and Membership-based models demonstrated better bootstrap apparent AUC performance compared to the other models, however, the Target-only model had a wider bootstrap test AUC CI, as shown in Figure S3 in Additional File. Additionally, both Intercept Recalibration models had smaller bootstrap test calibration slope CIs and lower mean calibration slope estimates than the other methods. However, both models showed wider CIs for the bootstrap apparent calibration slope.

### Performance of the modelling methods using existing development data

#### Internal validation in the target data: 1/1/2010–19/9/2012

**Fig. 6:**
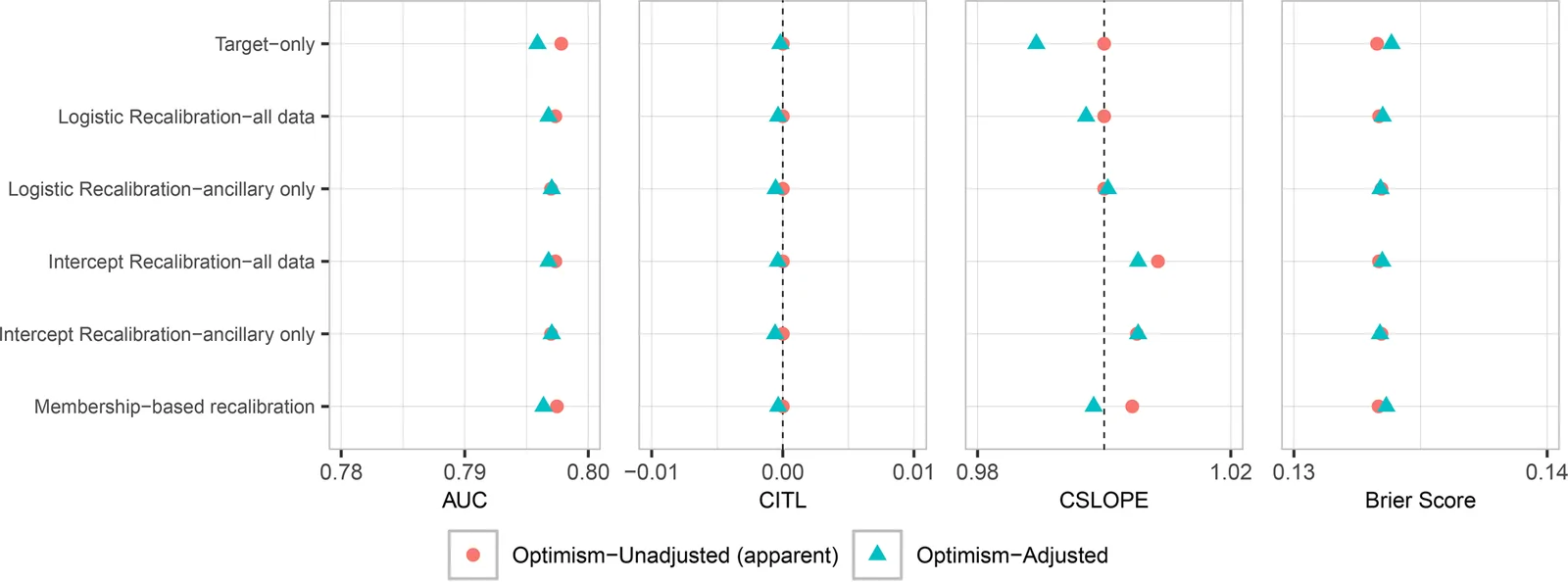
Internal validation-adjusted and unadjusted performance metrics: temporal–existing

For the optimism-adjusted internal validation in Fig. 6, all models showed similar performance, with AUC around 0.796–0.797 and Brier Scores near 0.133–0.134. Calibration-in-the-large was effectively zero, and calibration slopes were close to 1. Also, the Membership-based model had better bootstrap apparent and test AUC performance compared to the other models, as shown in Figure S4 in the Additional File. For the bootstrap test calibration slope, the Target-only model and both Logistic Recalibration models had wider CIs than the other models across 200 iterations. Conversely, for the bootstrap apparent calibration slope, both Intercept Recalibration models showed wider CIs.

#### External validation using data from the subsequent period: 20/9/2012–30/12/2014

**Fig. 7:**
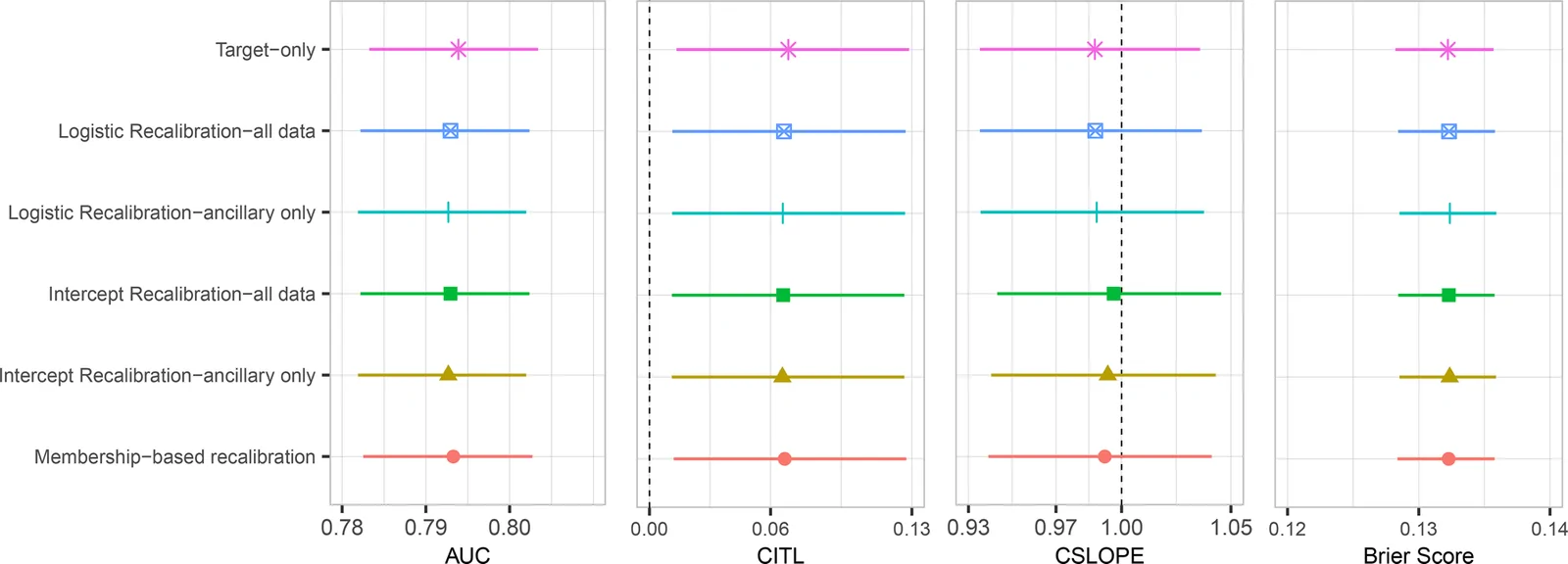
External validation for Temporal–existing *∙* candidate model external validation results on same target dataset are AUC: 0.793(0.782, 0.802), CITL: 0.283(0.228, 0.343), CSLOPE: 0.994(0.940, 1.043) , Brier score: 0.134(0.130, 0.138)

As shown in Fig. 7, all alternative models improved calibration-in-the-large relative to the candidate model, with mean CITL values ranging from 0.066 to 0.069 compared with 0.283 (0.228, 0.343) for the candidate model. In terms of the difference between expected and observed survival, as shown in Table 4, the candidate model overestimated risk (or underestimated survival) by 3.0 percentage points when externally validated using data from the subsequent period. In contrast, the alternative models underestimated survival by 0.8 to 0.9 percentage points. The lower calibration-in-the-large values of the alternative models compared with the candidate model (Table S7, supplementary material) suggest that applying the alternative methods during model development may have reduced the potential effect of temporal heterogeneity within the original development data. However, the observed differences may also reflect random variation or characteristics of the particular data analysed.

## Discussion

This study aimed to validate an existing COPD prognostic model across different temporal and regional settings and to assess various modelling methods for their potential to improve predictive performance by incorporating ancillary data while accounting for heterogeneity. Overall, the candidate model maintained similar discrimination across the validation settings, but calibration varied, emphasising the need for recalibration to address baseline risk shifts over time. Incorporating ancillary data through recalibration or membership-based methods generally improved model calibration, especially calibration-in-the-large, across the settings considered. Among the methods evaluated, Intercept Recalibration methods generally demonstrated consistent performance, often achieving optimism-adjusted calibration slopes closer to 1 and narrower confidence intervals in bootstrap test samples. Generally, applying Recalibration methods did not worsen the performance measures evaluated, even when recalibration may not have been strictly necessary, such as in the North East setting.

This aligns with a growing trend in the literature toward adapting risk prediction models to contemporary and geographically diverse populations. For example, the SCORE2 Working Group and the ESC Cardiovascular Risk Collaboration emphasised the necessity of regional recalibration for the SCORE2 cardiovascular risk model [33]. By incorporating updated incidence rates and regional risk factor distributions, they improved the model’s applicability across European populations. Importantly, they also highlighted limitations of the original SCORE model, which often misestimated risk in specific regions due to the lack of systematic recalibration to account for contemporary event rates and regional variation [33]. Other studies have similarly demonstrated the importance of adapting risk models to local settings when there is a heterogeneity between the target and ancillary populations. Kappen et al. [17] showed that logistic recalibration of existing models using local patient data significantly improved performance, outperforming both Kiddle et al.’s model and newly developed local models in terms of calibration. Janssen et al. [34] further supported this by showing that intercept recalibration is an effective method to improve models applicability to local populations when the incidence of the outcome differs across settings.

Evidence from other diverse populations reinforces this point, Hua et al. [35] found that the Framingham models underestimated cardiovascular risk in an Australian Indigenous cohort, but recalibration of the 2008 model improved performance, enabling development of a tailored risk chart. However, recalibration is not always the best solution, Yang et al. [36] evaluated a COPD risk prediction model originally developed in a Western population when applied to Chinese adults. They found that the model overestimated the risk of COPD among older individuals. Even after recalibration, the model continued to over-predict the 5-year risk for both genders. Building on this concept, incorporating the ancillary dataset is not limited to adapting an existing model to a new target setting. It can also be used proactively during model development for a particular target population, leveraging data from multiple time points/settings within a given development dataset to improve performance overall. In our analysis, applying the alternative methods during model development improved calibration, reducing the calibration-in-the-large to 0.06 and decreasing the difference between expected and observed outcomes from roughly 3 to 1 percentage points. This suggests that applying the alternative methods during model development may have reduced the potential effect of temporal heterogeneity within the original development data [25]. However, the observed differences may also reflect random variation or characteristics of the particular data analysed.

Supporting the importance of reassessing calibration over time, Ng et al. [37] recommended recalibrating the PROgnostic ModEl for chronic lung disease (PRO-MEL)—either by adjusting the intercept or by reweighting the predictors—to minimize prediction bias, given the evolving characteristics of patient populations over time. Puhan et al. [38] also advised recalibration after evaluating the performance of the BODE index for predicting 3-year mortality in COPD patients and observing poor calibration when the model was applied to external cohorts [7], with the BODE index underestimating mortality in a Swiss cohort and overestimating it in a Spanish cohort. All methods including Target-only, Membership-based, Intercept Recalibration, and Logistic Recalibration, generally had calibration-in-the-large estimates closer to zero across the settings considered. AUC improvements were modest for both Intercept and Logistic Recalibration methods, as expected, since these methods do not change the ranking of predicted risks across individuals. However, both methods often improved the calibration slope and Brier scores, indicating better overall predictive performance. Intercept Recalibration models generally had narrower bootstrap intervals for the calibration slope in test samples than the Logistic Recalibration models across the settings considered.

Across all settings except the North East region setting, the Target-only model demonstrated acceptable optimism-adjusted calibration slopes but it also showed wider bootstrap calibration slope confidence intervals in the test sample, similar to the Logistic Recalibration methods. The Membership-based recalibration model generally produced calibration estimates similar to those of the Recalibration methods and performed better than the Target-only model for some metrics. However, it had a lower calibration slope estimate and wider bootstrap test intervals in the North East setting. This may reflect instability associated with the effective sample size and the additional down-weighting of the ancillary data.

While this study provides important insights, several limitations should be acknowledged. First, the chosen prognostic model was published relatively recently. While the model was developed on patients diagnosed up to 20 years ago, it also included patients diagnosed as recently as 2012, meaning the total performance drift in the development population may not be as large compared to the most recent patient population as in other examples that were developed and published longer ago. A further limitation is the higher missingness of key variables (such as BMI and FEV1% predicted) in our reproduced analysis compared to Kiddle et al.’s model. This discrepancy may not just be due only to differences in data availability and structure but could also be unknown/other factors (such as differences in the data extraction methods), which restrict how closely we could replicate the original analysis. To remain consistent with the original Kiddle et al. model, we followed the published missing-data handling approach when reproducing the model. We acknowledge, however, that more advanced approaches, such as multiple imputation, may be preferable in practice [39, 40]. Additionally, since the model is recently published, it is not yet in use in clinical practice. This may slightly hinder the impact of the analysis compared to identifying and correcting performance drift in a well-used prognostic model. An additional consideration is that our study focused on validating an existing COPD model and evaluating different methods to improve model performance across temporal and regional settings; we did not specifically investigate the underlying drivers of miscalibration. In addition, although the original protocol considered multiple candidate models, the present analysis focused on Kiddle et al.’s model because it was the only model we were able to reproduce closely within our CPRD extract. Future work could explore the sources of calibration differences by first comparing regional differences within a fixed time period and then assessing temporal changes across regions. This may help identify whether observed miscalibration stems primarily from shifts in baseline risk, predictor distributions, or other regional and temporal factors.

Nonetheless, by systematically comparing multiple modelling methods, this study offers practical guidance on how to improve model calibration when applied to various target populations. This is particularly valuable in target datasets of limited sample size, where developing new models is often infeasible. Importantly, the evaluated methods can also be applied proactively during model development to account for potential calibration drift, reducing reliance on future data availability for updating efforts.

## Conclusion

This study suggests that incorporating ancillary data may enhance the calibration of a prognostic COPD model across temporal and regional settings. We evaluated performance using more recently collected data after model development and by leveraging the existing development dataset alone, showing that accounting for calibration drift during model development may allow a more up-to-date model to be developed from the outset in the presence of heterogeneity in the data. While all methods generally improved calibration-in-the-large, their performance varied across settings and performance metrics. Intercept and Logistic Recalibration methods generally demonstrated consistent performance, with Intercept Recalibration showing narrower bootstrap calibration slope intervals in test samples. The Target-only model showed acceptable calibration but greater variability in some settings, whereas the Membership-based method produced calibration estimates similar to the other methods in most settings, although it was sensitive to the very small target sample size in the North East region. Finally, Intercept Recalibration may provide a simple and useful approach, with the Membership-based Recalibration method providing a potential alternative.

## Electronic supplementary material

Below is the link to the electronic supplementary material.

Supplementary material 1

Supplementary material 2

Supplementary material 3

## Data Availability

The datasets analysed during the current study are not publicly available due to the required data sharing agreements and considerations. All statistical code for the analysis is available through open-source repositories (GitHub) for full transparency and reproducibility.

## Abbreviations

COPD: Chronic obstructive pulmonary disease
CITL: Calibration-in-the-large
BMI: Body mass index
FEV1% predicted: Forced expiratory volume in 1 second
CPRD: Clinical Practice Research Datalink
ONS: Office for National Statistics
HES: Hospital Episode Statistics
PPI: Patient and Public Involvement
CSLOPE: Calibration slope
CI: Confidence interval
