# Supplementary material for: Validation of a prognostic model for chronic obstructive pulmonary disease (COPD) and evaluation of methods to include related data
Source: Diagn Progn Res. 2026 Nov 6;10:31. doi: 10.1186/s41512-026-00245-8 (PMC13741443; doi:10.1186/s41512-026-00245-8)
Supplement: Supplementary file 1 [file 41512_2026_245_MOESM1_ESM.pdf]

S1 Cohort Replication Procedure and Differences from Kiddle et al. [1]

Full details of Kiddle study, cohort definition and methodology published here [1].

Patients were eligible if they were at least 35 years old, registered at a GP practice with up-to-standard data at the time of COPD diagnosis, and diagnosed between 1 January 2004 and 19 September 2012. We restricted to the CPRD 'linked group' (English practices consenting to linkage with ONS mortality and IMD data). Start and end dates for follow-up were defined as follows:

- Start ID = max of (year where patient is 35, up-to-standard date (uts), registration date (crd)).
- Start Date = max of (up-to-standard date (uts), registration date (crd)).
- End ID = min of (death date, transfer-out date (tod), last collection date (lcd)).

Patient inclusion rules were:

- COPD diagnosis date within study window (1st January 2004 to 19th September 2012)
- Start ID before COPD diagnosis date
- Start ID before End ID
- End ID after COPD diagnosis date
- Age is greater than or equal 35
- If the patient has at least a year of historical data between the Start Date of the study and COPD diagnosis date.

Variables were prepared as per the original model where possible. Continuous variables were median centred. Missing values in variables with a corresponding indicator of test not performed were set to the median observed value. Outliers were removed as follows: FEV1 above 5 litres, body mass index above 70 kg/m^2, eGFR above 200 mL/min/1.73m^3, c-reactive protein above 370 mg/L and albumin above 70 gL.

BMI was calculated as weight/$height^2$; the original publication did not specify whether BMI was derived from continuous measures or categorised medcodes, so both approaches were tested, but neither yielded identical missingness rates to those reported. Similarly, we used all FEV1% medcodes listed in Quint et al. [2], but the missingness rate remained higher than reported in Kiddle et al., likely reflecting differences in data extraction. All available information from the original paper and the accompanying GitHub repository was used. Despite this, unavoidable differences in data availability meant exact replication of the original cohort was not possible; our final cohort was therefore selected to match the original as closely as possible on inclusion criteria, study period, and baseline characteristics.

S2 Patient and Public Involvement

We discussed with public contributors how prognostic models may become out-of-date over time. For example, if the model is based on old data and there have been recent improvements in survival, this can lead to the risk predictions being too high for recently diagnosed patients. There were questions and discussion around how accurate prognostic models can be in practice and how often they are checked and updated. The general consensus from the group was that it is particularly important that prognostic models which predict serious health outcomes (e.g. mortality, having a heart attack, being diagnosed with cancer) are checked more regularly to ensure they are as accurate as possible.

S3 Results

Table S1: Adjusted and Unadjusted Performance Metrics (Internal Validation): Temporal–New

Model	Statistic	AUC	Calibration-in-the-large	Calibration slope	BrierScore
Membership-based recalibration	Optimism-Unadjusted (apparent)	0.796	0.000	1.005	0.132
	Optimism-Adjusted	0.795	-0.003	0.995	0.132
Intercept Recalibration-all data	Optimism-Unadjusted (apparent)	0.794	0.000	1.001	0.132
	Optimism-Adjusted	0.794	-0.003	0.999	0.132
Intercept Recalibration-ancillary only	Optimism-Unadjusted (apparent)	0.793	0.000	0.993	0.132
	Optimism-Adjusted	0.793	-0.003	0.994	0.133
Logistic Recalibration-all data	Optimism-Unadjusted (apparent)	0.794	0.000	1.000	0.132
	Optimism-Adjusted	0.794	-0.003	0.999	0.132
Logistic Recalibration-ancillary only	Optimism-Unadjusted (apparent)	0.793	0.000	1.000	0.132
	Optimism-Adjusted	0.793	-0.003	1.001	0.133
Target-only	Optimism-Unadjusted (apparent)	0.796	0.000	1.000	0.132
	Optimism-Adjusted	0.794	-0.003	0.985	0.133

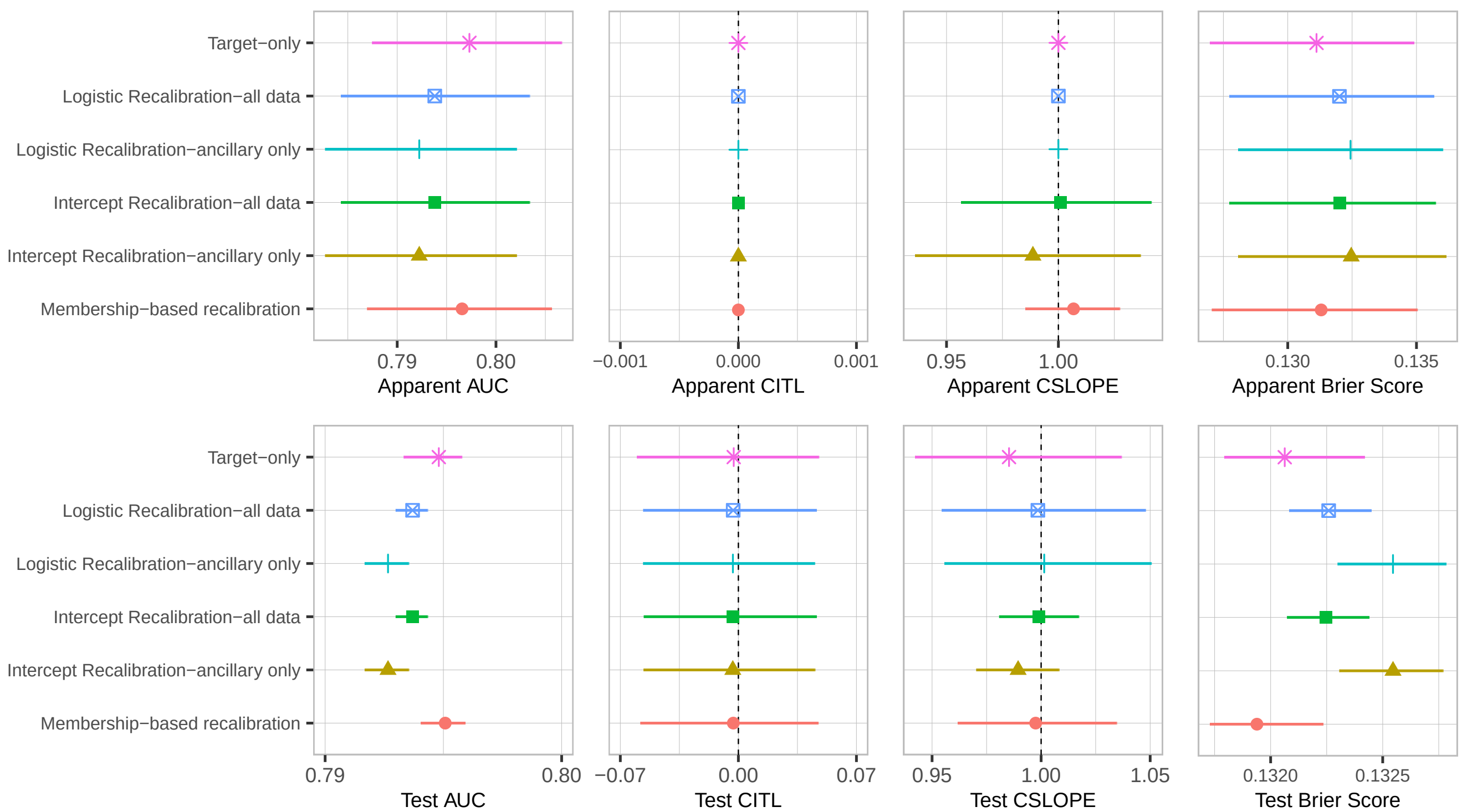

Figure S1: Bootstrap Performance Metrics' Confidence Intervals (Internal Validation): Temporal–New

Table S2: Bootstrap Performance Metrics' Confidence Intervals (Internal Validation): Temporal–New

Model	Statistic	AUC	Calibration-in-the-large	Calibration slope	Brier Score
Membership-based recalibration	Bootstrap Apparent	0.797(0.787, 0.806)	0(0, 0)	1.007(0.985, 1.027)	0.131(0.127, 0.135)
	Bootstrap Test	0.795(0.794, 0.796)	-0.003(-0.058, 0.047)	0.997(0.962, 1.035)	0.132(0.132, 0.132)
Intercept Recalibration-all data	Bootstrap Apparent	0.794(0.784, 0.803)	0(0, 0)	1.001(0.956, 1.041)	0.132(0.128, 0.136)
	Bootstrap Test	0.794(0.793, 0.794)	-0.003(-0.056, 0.046)	0.999(0.981, 1.017)	0.132(0.132, 0.132)
Intercept Recalibration-ancillary only	Bootstrap Apparent	0.792(0.783, 0.802)	0(0, 0)	0.989(0.936, 1.037)	0.132(0.128, 0.136)
	Bootstrap Test	0.793(0.792, 0.794)	-0.003(-0.056, 0.045)	0.989(0.970, 1.008)	0.133(0.132, 0.133)
Logistic Recalibration-all data	Bootstrap Apparent	0.794(0.784, 0.803)	0(0, 0)	1(1, 1)	0.132(0.128, 0.136)
	Bootstrap Test	0.794(0.793, 0.794)	-0.003(-0.056, 0.046)	0.999(0.954, 1.048)	0.132(0.132, 0.132)
Logistic Recalibration-ancillary only	Bootstrap Apparent	0.792(0.783, 0.802)	0(0, 0)	1(1, 1)	0.132(0.128, 0.136)
	Bootstrap Test	0.793(0.792, 0.794)	-0.003(-0.056, 0.045)	1.001(0.956, 1.050)	0.133(0.132, 0.133)
Target-only	Bootstrap Apparent	0.797(0.787, 0.807)	0(0, 0)	1(1, 1)	0.131(0.127, 0.135)
	Bootstrap Test	0.795(0.793, 0.796)	-0.003(-0.060, 0.048)	0.985(0.942, 1.037)	0.132(0.132, 0.132)

Table S3: Adjusted and Unadjusted Performance Metrics (Internal Validation): NorthEast–New

Model	Statistic	AUC	Calibration-in-the-large	Calibration slope	Brier Score
Membership-based recalibration	Optimism-Unadjusted (apparent)	0.786	0.000	0.989	0.146
	Optimism-Adjusted	0.753	-0.022	0.772	0.161
Intercept Recalibration-all data	Optimism-Unadjusted (apparent)	0.785	0.000	0.981	0.147
	Optimism-Adjusted	0.787	-0.019	0.975	0.148
Intercept Recalibration-ancillary only	Optimism-Unadjusted (apparent)	0.784	0.000	0.977	0.147
	Optimism-Adjusted	0.786	-0.019	0.974	0.148
Logistic Recalibration-all data	Optimism-Unadjusted (apparent)	0.785	0.000	1.000	0.147
	Optimism-Adjusted	0.787	-0.019	1.017	0.149
Logistic Recalibration-ancillary only	Optimism-Unadjusted (apparent)	0.784	0.000	1.000	0.147
	Optimism-Adjusted	0.786	-0.019	1.021	0.150
Target-only	Optimism-Unadjusted (apparent)	0.838	0.000	1.000	0.121
	Optimism-Adjusted	0.727	0.011	0.29	0.170

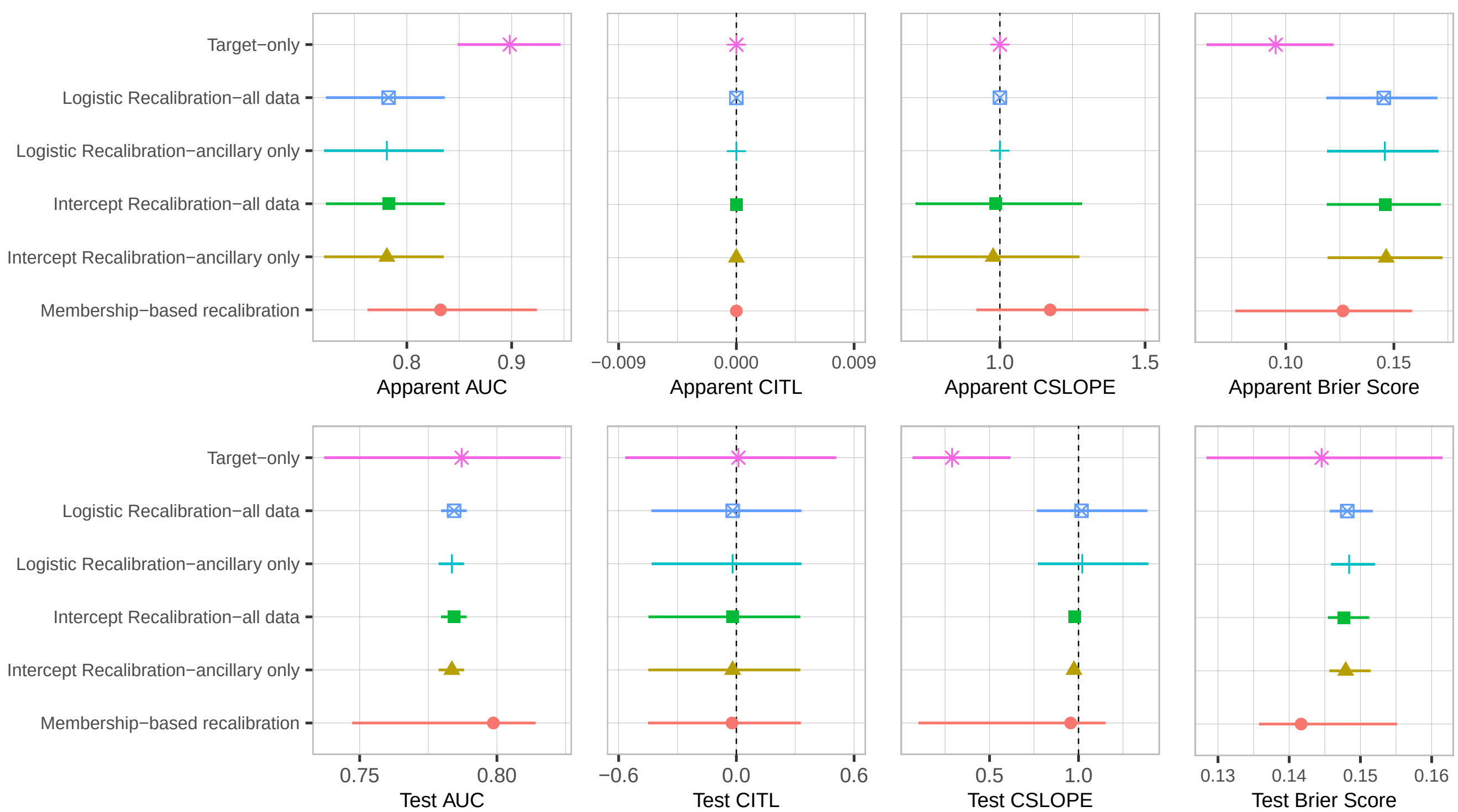

Figure S2: Bootstrap Performance Metrics' Confidence Intervals (Internal Validation): NorthEast–New

Table S4: Bootstrap Performance Metrics' Confidence Intervals (Internal Validation): NorthEast–New

Model	Statistic	AUC	Calibration-in-the-large	Calibration slope	Brier Score
Membership-based recalibration	Bootstrap Apparent	0.832(0.763, 0.924)	0(0, 0)	1.173(0.919, 1.510)	0.126(0.077, 0.158)
	Bootstrap Test	0.799(0.747, 0.814)	-0.022(-0.450, 0.327)	0.956(0.101, 1.150)	0.142(0.136, 0.155)
Intercept Recalibration-all data	Bootstrap Apparent	0.783(0.723, 0.836)	0(0, 0)	0.985(0.710, 1.281)	0.146(0.119, 0.172)
	Bootstrap Test	0.784(0.780, 0.789)	-0.019(-0.447 0.324)	0.979(0.950, 1.007)	0.148(0.145, 0.151)
Intercept Recalibration-ancillary only	Bootstrap Apparent	0.781(0.721, 0.835)	0(0, 0)	0.977(0.699, 1.273)	0.146(0.119, 0.172)
	Bootstrap Test	0.784(0.779, 0.788)	-0.019(-0.448, 0.324)	0.974(0.945, 1.003)	0.148(0.146, 0.151)
Logistic Recalibration-all data	Bootstrap Apparent	0.783(0.723, 0.836)	0(0, 0)	1(1, 1)	0.145(0.119, 0.170)
	Bootstrap Test	0.784(0.780, 0.789)	-0.019(-0.433, 0.330)	1.017(0.767, 1.385)	0.148(0.146, 0.152)
Logistic Recalibration-ancillary only	Bootstrap Apparent	0.781(0.721, 0.835)	0(0, 0)	1(1, 1)	0.146(0.119, 0.171)
	Bootstrap Test	0.784(0.779, 0.788)	-0.019(-0.431, 0.330)	1.021(0.772, 1.391)	0.148(0.146, 0.152)
Target-only	Bootstrap Apparent	0.898(0.849, 0.946)	0(0, 0)	1(1, 1)	0.095(0.063, 0.122)
	Bootstrap Test	0.787(0.737, 0.823)	0.011(-0.566, 0.507)	0.29(0.068, 0.615)	0.145(0.128, 0.161)

Table S5: Adjusted and Unadjusted Performance Metrics (Internal Validation): London–New

Model	Statistic	AUC	Calibration-in-the-large	Calibration slope	Brier Score
Membership-based recalibration	Optimism-Unadjusted (apparent)	0.810	0.000	0.977	0.121
	Optimism-Adjusted	0.799	-0.001	0.913	0.125
Intercept Recalibration-all data	Optimism-Unadjusted (apparent)	0.807	0.000	0.947	0.122
	Optimism-Adjusted	0.804	-0.001	0.935	0.123
Intercept Recalibration-ancillary only	Optimism-Unadjusted (apparent)	0.806	0.000	0.936	0.123
	Optimism-Adjusted	0.804	-0.002	0.928	0.123
Logistic Recalibration-all data	Optimism-Unadjusted (apparent)	0.807	0.000	1.000	0.122
	Optimism-Adjusted	0.804	-0.001	0.991	0.123
Logistic Recalibration-ancillary only	Optimism-Unadjusted (apparent)	0.806	0.000	1.000	0.122
	Optimism-Adjusted	0.804	-0.001	0.995	0.123
Target-only	Optimism-Unadjusted (apparent)	0.818	0.000	1.000	0.118
	Optimism-Adjusted	0.796	0.001	0.879	0.125

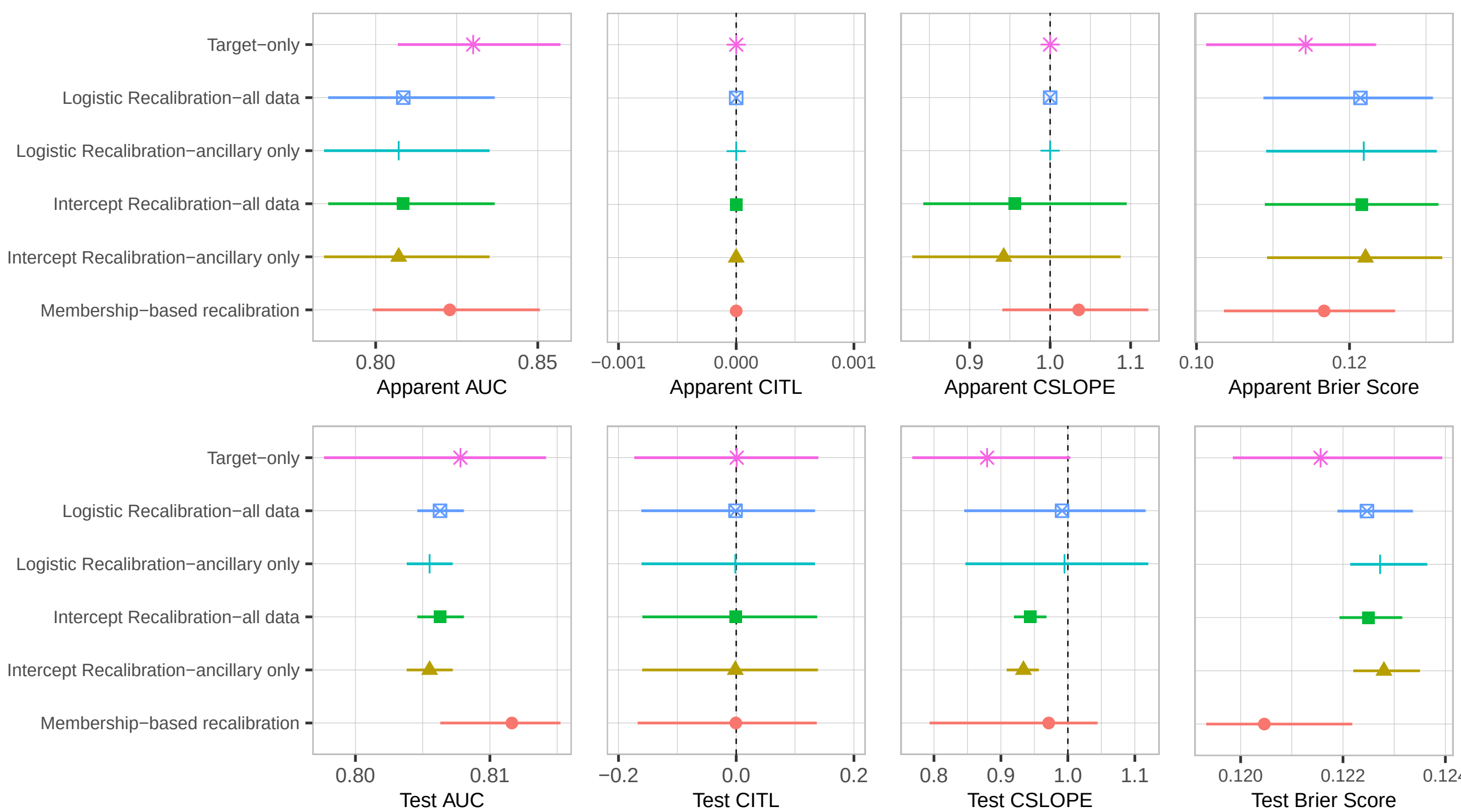

Figure S3: Bootstrap Performance Metrics' Confidence Intervals (Internal Validation): London–New

Table S6: Bootstrap Performance Metrics' Confidence Intervals (Internal Validation): London–New

Model	Statistic	AUC	Calibration-in-the-large	Calibration slope	Brier Score
Membership-based recalibration	Bootstrap Apparent	0.823(0.799, 0.851)	0(0, 0)	1.035(0.941, 1.121)	0.117(0.104, 0.126)
	Bootstrap Test	0.812(0.806, 0.815)	-0.001(-0.167, 0.136)	0.971(0.794, 1.044)	0.120(0.119, 0.122)
Intercept Recalibration-all data	Bootstrap Apparent	0.809(0.785, 0.837)	0(0, 0)	0.956(0.842, 1.094)	0.122(0.109, 0.132)
	Bootstrap Test	0.806(0.805, 0.808)	-0.001(-0.159, 0.137)	0.944(0.920, 0.967)	0.122(0.122, 0.123)
Intercept Recalibration-ancillary only	Bootstrap Apparent	0.807(0.784, 0.835)	0(0, 0)	0.942(0.829, 1.087)	0.122(0.109, 0.132)
	Bootstrap Test	0.806(0.804, 0.807)	-0.002(-0.160, 0.138)	0.934(0.909, 0.956)	0.123(0.122, 0.123)
Logistic Recalibration-all data	Bootstrap Apparent	0.809(0.785, 0.837)	0(0, 0)	1(1, 1)	0.121(0.109, 0.131)
	Bootstrap Test	0.806(0.805, 0.808)	-0.001(-0.161, 0.133)	0.991(0.845, 1.116)	0.122(0.122, 0.123)
Logistic Recalibration-ancillary only	Bootstrap Apparent	0.807(0.784, 0.835)	0(0, 0)	1(1, 1)	0.122(0.109, 0.131)
	Bootstrap Test	0.806(0.797, 0.799)	-0.001(-0.161, 0.133)	0.995(0.847, 1.119)	0.123(0.122, 0.124)
Target-only	Bootstrap Apparent	0.830(0.807, 0.857)	0(0, 0)	1(1, 1)	0.114(0.101, 0.123)
	Bootstrap Test	0.808(0.798, 0.814)	0.001(-0.173, 0.139)	0.879(0.768, 1.003)	0.122(0.120, 0.124)

Table S7: External Validation on data for patients diagnosed between 20/9/2012 to 30/12/2014 for "Temporal–Existing"

Model	AUC	Calibration-in-the-large	Calibration slope	Brier Score	Expected survival (%)	Difference in survival*
Membership-based recalibration	0.793(0.783, 0.803)	0.067(0.012, 0.127)	0.992(0.939, 1.041)	0.132(0.128, 0.136)	78%	-0.9%
Intercept Recalibration-all data	0.793(0.782, 0.802)	0.066(0.011, 0.126)	0.996(0.943, 1.046)	0.132(0.128, 0.136)	78%	-0.8%
Intercept Recalibration-ancillary only	0.793(0.782, 0.802)	0.066(0.011, 0.126)	0.994(0.940, 1.043)	0.132(0.129, 0.136)	78%	-0.8%
Logistic Recalibration-all data	0.793(0.782, 0.802)	0.067(0.011, 0.127)	0.988(0.935, 1.037)	0.132(0.128, 0.136)	78%	-0.8%
Logistic Recalibration-ancillary only	0.793(0.782, 0.802)	0.066(0.011, 0.127)	0.989(0.936, 1.038)	0.132(0.129, 0.136)	78%	-0.8%
Target-only	0.794(0.783, 0.803)	0.069(0.013, 0.129)	0.988(0.935, 1.036)	0.132(0.128, 0.136)	78%	-0.9%

*Difference between expected and observed survival. Observed survival is 79%. Expected survival for externally validated Candidate Model on same target dataset is 76%.

Table S8: Adjusted and Unadjusted Performance Metrics (Internal Validation): Temporal–Existing

Model	Statistic	AUC	Calibration-in-the-large	Calibration slope	Brier Score
Membership-based recalibration	Optimism-Unadjusted (apparent)	0.797	0.000	1.004	0.133
	Optimism-Adjusted	0.796	0.000	0.998	0.134
Intercept Recalibration-all data	Optimism-Unadjusted (apparent)	0.797	0.000	1.009	0.133
	Optimism-Adjusted	0.797	0.000	1.005	0.133
Intercept Recalibration-ancillary only	Optimism-Unadjusted (apparent)	0.797	0.000	1.005	0.133
	Optimism-Adjusted	0.797	-0.001	1.005	0.133
Logistic Recalibration-all data	Optimism-Unadjusted (apparent)	0.797	0.000	1.000	0.133
	Optimism-Adjusted	0.797	0.000	0.997	0.134
Logistic Recalibration-ancillary only	Optimism-Unadjusted (apparent)	0.797	0.000	1.000	0.133
	Optimism-Adjusted	0.797	-0.001	1.001	0.133
Target-only	Optimism-Unadjusted (apparent)	0.798	0.000	1.000	0.133
	Optimism-Adjusted	0.796	0.000	0.989	0.134

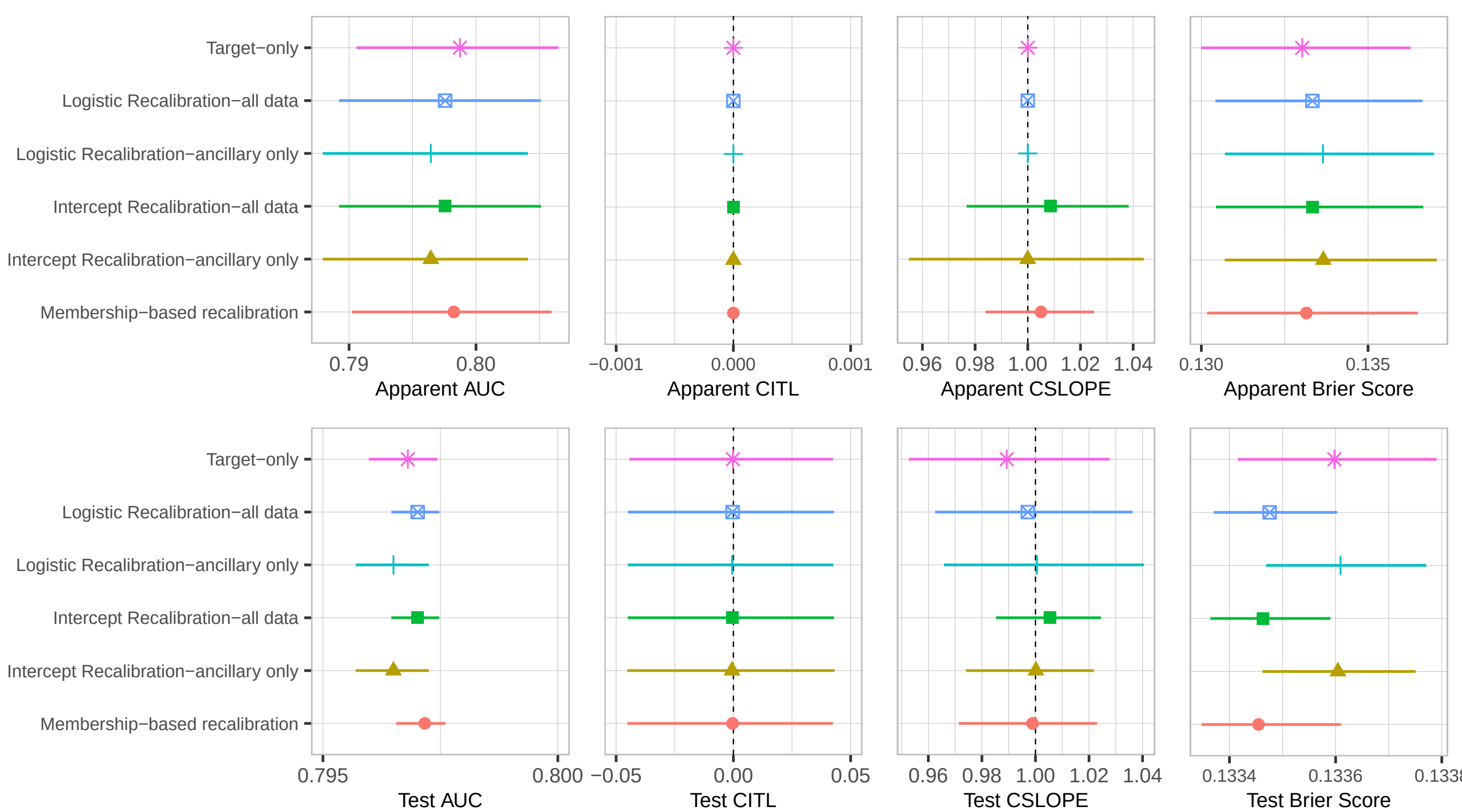

Figure S4: Bootstrap Performance Metrics' Confidence Intervals (Internal Validation): Temporal–Existing

Table S9: Bootstrap Performance Metrics' Confidence Intervals (Internal Validation): Temporal–Existing

Model	Statistic	AUC	Calibration-in-the-large	Calibration slope	Brier Score
Membership-based recalibration	Bootstrap Apparent	0.798(0.79,0.806)	0(0,0)	1.005(0.984,1.025)	0.133(0.13,0.136)
	Bootstrap Test	0.797(0.797,0.798)	0(-0.045,0.042)	0.999(0.972,1.023)	0.133(0.133,0.134)
Intercept Recalibration-all data	Bootstrap Apparent	0.798(0.789,0.805)	0(0,0)	1.009(0.977,1.038)	0.133(0.13,0.137)
	Bootstrap Test	0.797(0.796,0.797)	0(-0.045,0.043)	1.005(0.985,1.024)	0.133(0.133,0.134)
Intercept Recalibration-ancillary only	Bootstrap Apparent	0.796(0.788,0.804)	0(0,0)	1(0.955,1.044)	0.134(0.131,0.137)
	Bootstrap Test	0.797(0.796,0.797)	-0.001(-0.045,0.043)	1.001(0.974,1.022)	0.134(0.133,0.134)
Logistic Recalibration-all data	Bootstrap Apparent	0.798(0.789,0.805)	0(0,0)	1(1,1)	0.133(0.13,0.137)
	Bootstrap Test	0.797(0.796,0.797)	0(-0.045,0.043)	0.997(0.963,1.036)	0.133(0.133,0.134)
Logistic Recalibration-ancillary only	Bootstrap Apparent	0.796(0.788,0.804)	0(0,0)	1(1,1)	0.134(0.131,0.137)
	Bootstrap Test	0.797(0.796,0.797)	-0.001(-0.045,0.042)	1.001(0.966,1.04)	0.134(0.133,0.134)
Target-only	Bootstrap Apparent	0.799(0.791,0.806)	0(0,0)	1(1,1)	0.133(0.13,0.136)
	Bootstrap Test	0.797(0.796,0.797)	0(-0.044,0.042)	0.989(0.953,1.028)	0.134(0.133,0.134)

References

[1] S. J. Kiddle, H. R. Whittaker, S. R. Seaman, and J. K. Quint, "Prediction of five-year mortality after COPD diagnosis using primary care records," *PLoS One*, vol. 15, no. 7, p. e0236011, 2020.

[2] J. K. Quint, H. Müllerova, R. L. DiSantostefano, H. Forbes, S. Eaton, J. R. Hurst, K. Davis, and L. Smeeth, "Validation of chronic obstructive pulmonary disease recording in the clinical practice research datalink (CPRD-GOLD)," *BMJ open*, vol. 4, no. 7, p. e005540, 2014.
