# Supplementary material for: Validation of a prognostic model for chronic obstructive pulmonary disease (COPD) and evaluation of methods to include related data
Source: Diagn Progn Res. 2026 Nov 6;10:31. doi: 10.1186/s41512-026-00245-8 (PMC13741443; doi:10.1186/s41512-026-00245-8)
Supplement: Supplementary file 2 [file 41512_2026_245_MOESM2_ESM.pdf]

Medicines & Healthcare products Regulatory Agency

CPRD

1

General information

Protocol reference Id

23_003180

Study title

Validation of prognostic models for Chronic Obstructive Pulmonary Disease (COPD) and evaluation of methods to include related data to correct for miscalibration

Research Area

Methodological

Does this protocol describe an observational study using purely CPRD data?

Yes

Does this protocol involve requesting any additional information from GPs, or contact with patients?

No

2

Research team

**Role**	Chief Investigator
**Title**	Lecturer
**Full name**	Sarah Booth
**Affiliation/organisation**	University of Leicester
**Email**	sarah.booth@leicester.ac.uk
**Will this person be analysing the data?**	Yes
**Status**	Confirmed

**Role**	Corresponding Applicant
**Title**	Doctoral research fellow
**Full name**	Jonathan Broomfield
**Affiliation/organisation**	University of Leicester
**Email**	jb781@le.ac.uk
**Will this person be analysing the data?**	Yes
**Status**	Confirmed

**Role**	Collaborator
**Title**	Postgraduate
**Full name**	Haya Elayan
**Affiliation/organisation**	University of Manchester
**Email**	haya.elayan@postgrad.manchester.ac.uk
**Will this person be analysing the data?**	No
**Status**	Confirmed

**Role**	Collaborator
**Title**	Visiting Research Student
**Full name**	Haya Elayan
**Affiliation/organisation**	University of Leicester
**Email**	he63@leicester.ac.uk
**Will this person be analysing the data?**	Yes
**Status**	Confirmed

**Role**	Collaborator
**Title**	Lecturer
**Full name**	David Jenkins
**Affiliation/organisation**	University of Manchester
**Email**	david.jenkins-5@manchester.ac.uk
**Will this person be analysing the data?**	No
**Status**	Confirmed

**Role**	Collaborator
**Title**	Associate Professor
**Full name**	Mark Rutherford
**Affiliation/organisation**	University of Leicester
**Email**	mark.rutherford@le.ac.uk
**Will this person be analysing the data?**	Yes
**Status**	Confirmed

3

Access to data

Sponsor

University of Leicester

Funding source for the study

Is the funding source for the study the same as Chief Investigator's affiliation?

Yes

Funding source for the study

University of Leicester

Institution conducting the research

Is the institution conducting the research the same as Chief Investigator's affiliation?

Yes

Institution conducting the research

University of Leicester

Method to access the data

Indicate the method that will be used to access the data

Institutional multi-study licence

Is the institution the same as Chief Investigator's affiliation?

Yes

Institution name

University of Leicester

Extraction by CPRD

Will the dataset be extracted by CPRD

No

Multiple data delivery

This study requires multiple data extractions over its lifespan

No

Data processors

**Data processor is**	Same as the chief investigator's affiliation
**Processing**	Yes
**Accessing**	Yes
**Storing**	Yes
**Processing area**	UK

4

Information on data

Primary care data

CPRD GOLD

Do you require data linkages
Yes

Patient level data

HES Admitted Patient Care
ONS Death Registration Data

NCRAS data

Covid 19 linkages

Area level data
Do you require area level data?
Yes

Practice level (UK)

Patient level (England only)

Patient Level Index of Multiple Deprivation

Withheld concepts

Are withheld concepts required?

No

Linkage to a dataset not listed

Are you requesting a linkage to a dataset not listed?

No

Patient data privacy

Does any person named in this application already have access to any of these data in a patient identifiable form, or associated with an identifiable patient index?

No

5

Protocol information

Lay Summary

Prognostic models estimate future outcomes or risks associated with a particular disease for an individual patient. These models use various patient characteristics (like age and smoking status) and clinical data (like blood pressure) to generate predictions about a patient's prognosis. These predictions are used by healthcare professionals in treatment planning, particularly for chronic diseases where predicting the course of the illness can help guide interventions and improve patient outcomes.

One such chronic disease for which many prognostic models have been developed is chronic obstructive pulmonary disease (COPD), a progressive lung disease affecting the ability to breathe. Provided a diagnosis is made early, patients can live for 10 to 20 years. Models have been developed to predict the risk of death up to 10 years after diagnosis, meaning that patients from 10 to 20 years ago are included in the model. However, survival both in the disease area and in the general population may have improved since then. This means that by the time the model is published it may already be, or soon become, out of date. In addition, survival may vary by geographical region which could lead to prognostic models giving less accurate predictions for patients living in particular areas.

We will evaluate three existing COPD prognostic models and assess their performance in the current UK population. If the predictions are out of date or less accurate for patients living in certain regions, we will evaluate methods that can be used to update these models and improve their accuracy.

Technical Summary

Prognostic models are statistical algorithms that predict the risk of future health outcomes. They are usually developed using logistic or survival regression and use patient characteristics and clinical data to make predictions. These predictions are used by healthcare professionals in treatment prescriptions and disease management.

Many prognostic models that predict mortality have been developed for chronic obstructive pulmonary disease (COPD), a progressive lung disease that is particularly prevalent in smokers. Producing long-term predictions requires long-term follow-up and means that patients diagnosed many years ago have to be included. However, if survival outcomes have improved, the risk for these patients may be different to more recently diagnosed patients, which leads to model miscalibration.

Using recent data, we will temporally validate three prognostic models for COPD mortality to evaluate the drift of these models over time, assess the need for recalibration, and if required, recalibrate them for the current UK population.

Period analysis can be used for time-to-event prognostic modelling by using more recent cohorts, but this decreases the sample size which can lead to overfitting. Instead, we will evaluate newly developed methods including temporal recalibration and weighted regression and compare these to standard model updating approaches (e.g. updating the baseline and calibration slope). In temporal recalibration, the predictor effects from the full cohort are preserved while baseline mortality is updated based on a period analysis, giving up-to-date recalibrated predictions that utilise the power of the full sample size. A weighted regression approach accounts for the case-mix to investigate model recalibration by re-weighting the data according to the observed distribution shift.

We will also investigate calibration by geographical region since miscalibration can occur if the baseline mortality risk or case-mix differs across the UK. If miscalibrated, we will explore model updating and weighting approaches to improve the prediction accuracy for specific regions.

Outcomes to be measured

Mortality (at 1, 5 and 10 years of follow-up following COPD diagnosis)

Objectives, specific aims & rationale

Objective: to assess calibration drift in prognostic models for COPD and variation in calibration by geographical region, and evaluate whether this miscalibration can be fixed with temporal recalibration or other model updating strategies.

Specific Aims: to evaluate and update three specific prognostic models (1) - (3), where survival may have improved since follow-up from these studies started and where there may be variation in mortality or case-mix by geographical region.

Rationale: Absolute risk estimates must be accurate since treatment strategies often depend directly upon published prognostic models. These models can quickly become inaccurate and overestimate the risk of a patient experiencing an event if survival or standards of care improve in the disease area. Risk predictions may also be inaccurate for patients living in certain regions if there is variation in survival or case-mix across the UK.

Study background

Chronic Obstructive Pulmonary Disease (COPD) is a progressive lung disease that primarily affects the ability to breathe. It is a long-term condition characterized by persistent airflow limitation and inflammation of the airways (4). The prevalence of COPD in the UK is estimated to be around 1-2% (5).

The symptoms of COPD often develop gradually and worsen over time. They include shortness of breath, chronic cough, wheezing, chest tightness, frequent respiratory infections, fatigue, and unintended weight loss. As the condition progresses, everyday activities become increasingly difficult, impacting a patient's quality of life.

Diagnosis of COPD involves medical history evaluation, physical examination, lung function tests like spirometry, and imaging studies. Treatment focuses on managing symptoms, preventing exacerbations, and slowing down the progression of the disease. It typically involves a combination of medications (bronchodilators, steroids), pulmonary rehabilitation, oxygen therapy, and lifestyle changes such as smoking cessation and avoiding lung irritants.

Prognostic models aim to predict the risk of an event occurring in a particular population with a disease, based on relevant clinical information (6) (7). Prognostic models for COPD therefore often include usual predictors of health differences, such as age, gender and BMI, alongside disease-specific predictors like forced expiratory volume in 1 second (FEV1). Three prognostic models are considered in this study that all used the CPRD to identify patients and develop the models; the outcomes were mortality at 1 year (1), 5 years (2) and 10 years (3). Models such as these can be used by health practitioners to administer care, assist with disease planning and management and (in some instances) prescribe treatments to patients at an elevated risk.

However, for models with longer-term outcomes, such as 5- or 10-year mortality, extended follow-up is required such that patients diagnosed a number of years ago are included in model development. If survival has improved in this time, this can cause the model to give miscalibrated predictions that overestimate the risk of dying in more recent populations. One solution to this problem is to conduct a period analysis on the most recent cohort, meaning that only the risk-time that patients experience during this recent period contributes to the analysis (8). This approach has been used in prognostic modelling (9) but has limitations, as the sample size is drastically reduced which can cause overfitting during model development.

A recently proposed alternative by the authors is temporal recalibration (10), which combines period analysis with recalibration methods. The full dataset is used to estimate the predictor effects and the baseline is recalibrated to a more recent time period through period analysis. This approach has been assessed in the development of prognostic models, and through this study will be assessed as an approach to update existing prognostic models to current patient populations. The study will investigate the need to temporally update prognostic models to populations of interest. Another alternative for logistic regression is through weighted regression analysis, where a weighted dataset is constructed to resemble the distribution of the current patient population and thus recalibrate the prognostic model. These approaches will also be compared to standard model updating methods (e.g. updating the baseline, updating the calibration slope).

A further issue with prognostic models is that if there is variation in survival or case-mix by geographical region, not accounting for this can lead to miscalibrated predictions for patients living in particular regions. If there is miscalibration, the application of standard model updating methods (e.g. updating the baseline, updating the calibration slope) and the weighted regression approach will be explored for improving the calibration of the predictions for patients living in particular areas.

Study type

This is a methodological study validating and updating existing prognostic models through methods such as temporal recalibration (10) and weighted regression.

Study design

This is a retrospective, longitudinal cohort study.

Feasibility counts

A feasibility count in CPRD GOLD identified a total 395,934 patients diagnosed with COPD, of which 163,201 had a diagnosis after 26/06/2015, when the most recent study's follow-up ended (3) - these patients will be used in the temporal recalibration of the models. Diagnosis was determined using previously validated COPD codes (11) which are given in the Appendix.

The three prognostic models that are being validated also used CPRD GOLD data for model development, and respectively identified 54,990 (1), 47,964 (2) and 37,485 (3) COPD patients in their studies.

Sample size considerations

The existing prognostic models were developed using the CPRD, and suitable patient numbers were derived to estimate prognostic scores from. This study will use the same patients (as well as more recent data since publication of the models) to recalibrate the models through temporal recalibration, which maximises the sample size.

Planned use of linked data and benefit to patients in England and Wales

Linking to ONS data for this study will provide access to (more accurate) date of death and replicate the analysis in the development of the prognostic models. Linking to HES is required to identify some covariates included in the prognostic models, while patient-level IMD allows socio-economic status for patients to be determined (also included in the models).

By assessing the methods for temporal validation and recalibration of prognostic models, this study will improve the accuracy, not just of the discussed prognostic models, but of any models. This will lead to direct improvements in patient care through the implementation of these more accurate prognostic models in clinical practice in England and Wales.

Definition of the study population

The study population is any patient aged 35 or over in CPRD GOLD diagnosed with COPD. Diagnosis codes are taken from a previous validation study of the codes (11) and included in the Appendix. Patients will be required to have a diagnosis of COPD in their CPRD records and be aged 35 or over, since this is consistent with two of the previous prognostic models (2-3) and clinical opinion. Patient data will be included in the analysis following the latest of their diagnosis date, their registration date with CPRD, the cohort start date and the date their practice began recording research quality data.

In the main analysis, only patients that are eligible for linkage to HES will be included, since some covariate definitions are reliant upon this data. We will investigate the comparability of the linked and unlinked populations in a sensitivity analysis and compare results between the two based on covariates that can only be determined using CPRD data. We will also relax the diagnosis criteria from patients aged 35 and over to patients of any age with a COPD diagnosis to replicate the criteria used in Bloom et al's study (1) in a sensitivity analysis. When reconstructing the models, we will use the same definitions and inclusion/exclusion criteria as the original studies.

Selection of comparison groups/controls

No controls are required since the prognostic models only determine the risk of mortality in patients diagnosed with COPD.

Exposures, outcomes and covariates

The exposure is diagnosis of COPD (defined earlier), and the outcome is mortality at 1, 5 and 10 years from diagnosis (determined from ONS, or CPRD records if ONS is missing). Covariates are determined from the covariates considered by the existing prognostic models, and so their extraction is feasible and any issues can be resolved by contacting the authors of the prognostic models. The following covariates will be extracted from CPRD and HES records (either taken as close as possible to the date of COPD diagnosis or, if handled as a time varying covariate in the model, at each instance of recording over follow-up):

Continuous:
• Age - available from Clinical file
o Exact date of birth is imputed at halfway points where these are not available i.e. 15/month/year if patients are <= 16 and 1/7/year if patients are > 16
o If patients have a record appearing earlier in the year than these imputed dates then that date will be taken as date of birth instead
• BMI - weight (kg)/height (m)^2, available from Clinical file
• Creatinine - available from Test file
• Eosinophil - available from Test file
• FEV1 - determined by spirometry values, available from Test file
• Haemoglobin - available from Test file

Categorical:
• Acute respiratory infection history - available from Clinical file
• Albumin - low/high (35 g/L cut-off), available from Test file
• Alcohol problems - available from Clinical file
• Anaemia - available from Clinical file
• Anxiety - available from Clinical file
• Asthma - available from Clinical file
• Atrial fibrillation - available from Clinical file
• Bereavement - available from Clinical file
• Breathlessness - available from Clinical file
• Cancer - available from Clinical file
• Chronic Kidney Disease - available from Clinical file
• Connective tissue disorders - available from Clinical file
• Constipation - available from Treatment file (4 or more laxative prescriptions in last year)
• Coronary artery disease - available from Clinical file
• C-reactive protein - available from Test file
• Dementia - available from Clinical file
• Depression - available from Clinical file
• Diabetes - available from Clinical file
• DOSE index - determined by MRC, FEV1, smoking and exacerbations, available from Test/Clinical file
• Epilepsy - available from Clinical file
• Family history of respiratory diseases - available from Clinical file
• Fibrinogen - available from Test file
• Gastric ulcer disease - available from Clinical file
• Gastro-oesophageal reflux disease - available from Clinical file
• Gender - available from Clinical file
• GOLD stage - determined by FEV1 and FVC, available from Test file
• Heart failure - available from Clinical file
• Hospitalisations - number of hospitalisations per year, available from HES files
• Hypertension - available from Clinical file

- Ischaemic heart disease - available from Clinical file
- IBS - available from Clinical file
- Inflammatory bowel disease - available from Clinical file
- Influenza vaccinations - available from Clinical file
- Inhaled corticosteroid use - available from Therapy file
- Liver disease - available from Clinical file
- Long acting beta agonist/muscarinic antagonist use - available from Therapy file
- Lung fibrosis - defined as any interstitial lung disease, available from Clinical file
- (Modified) MRC dyspnoea score - available from Clinical file
- Myocardial infarction - available from Clinical file
- Number of exacerbations (0, 1-2 hospitalisations, >=3), available from HES files
- Osteoarthritis - available from Clinical file
- Osteoporosis - available from Clinical file
- Oxygen therapy - available from Clinical file
- Peripheral vascular disorder - available from Clinical file
- Platelets - low/normal/high (cut-offs at 150 and 400 x109 /L), available from Test file
- Pneumococcal vaccinations - available from Clinical file
- Pulmonary embolism - available from Clinical file
- Pulmonary fibrosis - available from Clinical file
- Pulmonary tuberculosis - available from Clinical file
- Psychosis/bipolar - available from Clinical file
- Rheumatoid arthritis - available from Clinical file
- Socioeconomic status - available from linked Patient Level IMD data
- Smoking status - available from Clinical file, most recently available
- Stroke - available from Clinical file
- Substance abuse - available from Clinical file
- Ventilation use - available from Clinical file and HES records

The majority of these are typical clinical predictors which the researchers have experience extracting, and for which there is much published guidance and examples (such as the CPRD @

Cambridge published code lists (12)). For more disease-specific predictors, such as creatinine and haemoglobin, the methods for extraction will be mirrored from the original papers (since these details were provided) to ensure the appropriate codes and scores will be used.

Data/statistical analysis

In order to assess calibration of the previous models in more recent data, we will reconstruct the models. While covariates (and their functional forms) in each of these previous models were developed using a combination of clinical and statistical approaches, we will just be replicating these models. Unless otherwise stated, continuous covariates are included linearly and categorical covariates as a collection of indicator variables.

Bloom et al developed a Cox survival model to predict 1-year mortality (1) based on the 12 comorbidities used in the COTE index, depression, diabetes, pulmonary embolism blood test data and bereavement status. Kiddle et al developed a logistic regression model predicting 5-year mortality (2) based on age, BMI and forced expiratory volume (all modelled using a linear and quadratic term), sex, smoking status, alcohol problems, atrial fibrillation, diabetes, heart failure, inflammatory bowel disease, peripheral vascular disorder, substance abuse, connective tissue disorders, stroke, asthma, cancer, constipation, depression, epilepsy, irritable bowel syndrome and psychosis/bipolar. Shah et al constructed a Cox survival model for 10-year mortality (3) based on age, sex, smoking status (modelled using a finite state model to account for changes over time), pulmonary tuberculosis, exercise, occupational exposure, physical activity, ischaemic heart disease, deprivation status, MRC scale, exacerbations, breathlessness, acute respiratory infections, anxiety, depression, family history of respiratory diseases, heart failure and hypertension.

We will first temporally validate each of these models to assess the accuracy of the predictions for more recently diagnosed patients. We will estimate calibration-in-the-large using the O/E ratio, which compares the observed risk of death (O) to the average predicted risk (p_i) across all individuals in the validation dataset (E=1/N _(i=1)^N p_i) at the time point of interest (1, 5 or 10 years). We will also assess calibration in more detail by producing a calibration plot for each model and calculating the calibration slope. This will be used to assess the agreement between the predicted and observed risks over different risk groups and show whether certain risk groups are more susceptible to under- or over- predictions of risk from the model.

Whilst the main focus will be assessing the calibration of the risk predictions, measures of discrimination will also be calculated in the more recent study cohort and compared to the reported measures from the original studies. Other metrics used to assess the performance of the models will include the AUC and Brier Score.

If there is evidence of calibration drift, we will compare a range of methods for updating these models to account for the changes in the survival outcome. Commonly used methods for model updating will be investigated and for the logistic regression model, this will consist of re-estimating the baseline (intercept) whilst fixing the linear predictor terms, re-estimating the baseline and scaling the linear predictor, and re-estimating the baseline and some/all of the covariate effects included in the linear predictor. Coefficients which may have changed since the original model publications will be re-estimated or removed if they are no longer relevant, while some new clinical predictors that were not considered in the models will also be investigated. The equivalent approach of each method will be taken for the survival models (re-estimating the baseline hazard function rather than the intercept term). These models will be internally validated using bootstrapping and optimism-adjusted performance metrics will be calculated.

We will also compare two novel methods to account for calibration drift; temporal recalibration and a weighted regression approach. The methods of temporal recalibration are detailed elsewhere (10) and explained briefly here. The first stage will be to replicate the development of the three identified prognostic models, which will involve defining covariates in the same way as the original studies. Then, the survival or logistic regression model will be fitted with the covariate effects constrained to be equal to the observed effects from the published models and the baseline will

be re-estimated using a subset of the data from the population/timeframe of interest. For logistic regression models this will only include those diagnosed in the most recent years and for survival models this will be defined using a period analysis sample. This recalibration gives up-to-date predictions without the need to estimate covariate effects only from the period analysis or recently diagnosed sample.

The models will also be assessed for miscalibration and updated through weighted regression based on newly developed data driven weighting methods to account for the case-mix shift in the dataset using distance metric and membership (probabilistic) metric. These two methods re-weight data in such a way as to correct for distributional changes, thus improving the model fit for the latest distribution shift in the dataset that reflects the distribution of the population where the model is intended to make predictions or estimate risks. The approaches produce a set of weights that are used to construct a weighted dataset that resembles the distribution of the most recent dataset, by giving the data samples higher weights when they are more relevant to the most recent dataset and lower weights when they are less relevant. This is done by measuring the relatedness of the data samples compared to most recent dataset then adjusting the distribution of the dataset to represent the distribution of the most recent dataset. For the membership-based weighted model, the baseline can then be recalibrated by including an additional binary predictor in the model which indicates whether the sample was from the original or more recent data.

The one-year, five-year and ten-year mortality predictions from the updated prognostic models will again be assessed using performance metrics such as the O/E ratio and calibration plots. They will also be compared to those from the original prognostic models to assess the impact of temporal recalibration and other model updating methods.

Shah et al did not report any baseline survival estimate in their model (3), and so we will reconstruct their final model exactly (such as by including data only prior to May 2011) to obtain the baseline survival term that would have been observed in the reported prognostic model, allowing calibration for this model to be assessed.

A further issue in prognostic modelling is that if there is variation in survival or case-mix across geographical regions, this can lead to miscalibrated predictions for patients living in particular areas. The calibration of the predictions by region will also be assessed. If there is miscalibration,

model updating methods (e.g. updating the slope, updating the calibration slope) and weighted regression approaches will be assessed as strategies for improving the calibration for these regions.

Plan for addressing confounding

Confounding is not considered here because we are not investigating how differences in exposures affect outcomes.

Plans for addressing missing data

Date of birth will be imputed as described in Section M. A sensitivity analysis will vary these assumptions to impute date of birth at the start of the month (patients aged <= 16) or year (patients aged > 16) of birth that is given.

Covariate information was fairly well recorded in the previous prognostic models that we are investigating (1)-(3). In some instances, large proportions of missing data were observed for covariates in the prognostic models (such as for FEV1 % predicted, BMI and MRC dyspnoea score) - in these instances, dichotomous covariates were included in the models indicating whether or not a patient had a missing covariate value. The approach for modelling the missing value then varied from model to model; multiple imputation (1), complete case analysis (2) and median-observed-value imputation (3) approaches were adopted. We will replicate these approaches for each model in turn in our analysis, and use multiple imputation when validating the models according to published recommendations (13).

Patient or user group involvement

We will consult with a COPD-specific patient group to determine at what point model miscalibration becomes problematic for them, i.e. what magnitude of bias is manageable and what would be the cut-off for the change in risk level that would mean a model requires updating. This is particularly important due to the use of prognostic modelling in disease planning and management between patients and health experts.

Plans for disseminating & communicating

The temporally and regionally recalibrated prognostic models will be published in a paper detailing the motivation behind recalibration. This paper will report the temporal validation of the prognostic models and highlight the problems that can arise when using prognostic models that were developed in a population that no longer has the same disease progression as the population of interest. The approaches of temporal recalibration and weighted regression will be demonstrated for other researchers. The results of the analysis will also be presented at an international conference and at external seminars, including at the University of Manchester.

Conflict of interest statement

None to declare.

Limitations of study design

The three prognostic models that have been chosen were all published relatively recently. While the models were developed on patients diagnosed up to 30 years ago, they also included patients diagnosed as recently as 2015, meaning the total calibration drift in the development population may not be as large compared to the most recent patient population as in other examples that were developed and published longer ago. However, there will still be some drift between the two populations, and the analysis will serve as a demonstration of the importance of accounting for any drift that may have occurred.

Additionally, since the models are recently published, they are not yet in use in clinical practice. This may slightly hinder the impact of the analysis compared to identifying and correcting calibration drift in a well-used prognostic model.

References

(1) Bloom CI, Ricciardi F, Smeeth L et al. Predicting COPD 1-year mortality using prognostic predictors routinely measured in primary care. BMC Med 2019;17:73.
(2) Kiddle SJ, Whittaker HR, Seaman SR and Quint JK. Prediction of five-year mortality after COPD diagnosis using primary care records. PLoS ONE 2020;15(7):e0236011.
(3) Shah SA, Nwaru BI, Sheikh A et al. Development and validation of a multivariable mortality risk prediction model for COPD in primary care. NPJ Prim Care Respir Med 2022;32:21.
(4) Vestbo J, Hurd SS, Agustí AG et al. Global strategy for the diagnosis, management, and prevention of chronic obstructive pulmonary disease: GOLD executive summary. Am J Respir Crit Care Med 2013; 187:347-365.
(5) Calverley P, Bellamy D. The challenge of providing better care for patients with chronic obstructive pulmonary disease: the poor relation of airways obstruction? Thorax 2000; 55: 78-82.
(6) Riley RD, van der Windt D, Croft P, Moons K. Prognosis Research in Healthcare: Concepts, Methods, and Impact. Oxford: Oxford University Press, 2019.
(7) Steyerberg EW, Moons KG, van der Windt DA et al; for the PROGRESS Group. Prognosis Research Strategy (PROGRESS) 3: prognostic model research. PLoS Med 2013; 10:e1001381.
(8) Smith LK, Lambert PC, Botha JL, Jones DR. Providing more up-to-date estimates of patient survival: a comparison of standard survival analysis with period analysis using life-table methods and proportional hazards models. J Clin Epidemiol 2004; 57:14-20.
(9) Keogh RH, Szczesniak R, Taylor-Robinson D, Bilton D. Up-to-date and projected estimates of survival for people with cystic fibrosis using baseline characteristics: a longitudinal study using UK patient registry data. J Cyst Fibros 2018; 17:218-227.
(10) Booth S, Riley RD, Ensor J, Lambert PC, Rutherford MJ. Temporal recalibration for improving prognostic model development and risk predictions in settings where survival is improving over time. Int J Epidemiol 2020; 49(4):1316-1325.
(11) Quint JK, Mullerova H, DiSantostefano RL et al. Validation of chronic obstructive pulmonary disease recording in the clinical practice research datalink (CPRD-GOLD). BMJ Open 2014;4:e005540.
(12) CPRD @ Cambridge. Codes Lists (GOLD) Primary Care Unit 2020. Available at: https://www.phpc.cam.ac.uk/pcu/research/research-groups/crmh/cprd_cam/codelists/v11/ (Accessed: 25 July 2023).
(13) Tsvetanova A, Sperrin M, Peek N, Buchan I, Hyland S, Martin GP. Missing data was handled inconsistently in UK prediction models: a review of method used. J Clin Epidemiol. 2021;140:149-158.

Appendices

cprd-isac-appendix_0.pdf

Grant ID
